# Supplementary material for: Inducible cell-specific mouse models for paired epigenetic and transcriptomic studies of microglia and astroglia
Source: Commun Biol. 2020 Nov 19;3:693. doi: 10.1038/s42003-020-01418-x (PMC7678837; doi:10.1038/s42003-020-01418-x)
Supplement: Supplementary file 1 — Supplementary Information [file 42003_2020_1418_MOESM1_ESM.pdf]

Supplementary information

Inducible cell-specific mouse models for paired epigenetic and transcriptomic studies of microglia and astroglia

Authors: Ana J. Chucair-Elliott[1 §], Sarah R. Ocañas[1,2 §], David R. Stanford[1], Victor A. Ansere[1,2], Kyla B. Buettner[1,2], Hunter Porter[1,3], Nicole L. Eliason[4], Justin J. Reid [5], Amanda L. Sharpe[4], Michael B. Stout[6], Michael J. Beckstead[5], Benjamin F. Miller[5], Arlan Richardson[7,8], Willard M. Freeman[1,7,8*]

[1]Genes & Human Disease Program, Oklahoma Medical Research Foundation, Oklahoma City, OK USA, [2]Department of Physiology, University of Oklahoma Health Sciences Center, Oklahoma City, OK USA, [3]Oklahoma Center for Neuroscience, University of Oklahoma Health Sciences Center, Oklahoma City, OK USA, [4]Department of Pharmaceutical Sciences, University of Oklahoma Health Sciences Center, Oklahoma City, OK USA, [5]Aging & Metabolism Program, Oklahoma Medical Research Foundation, Oklahoma City, OK USA, [6]Department of Nutritional Sciences, University of Oklahoma Health Sciences Center, Oklahoma City, OK USA,[7]Department of Biochemistry, University of Oklahoma Health Sciences Center, Oklahoma City, OK, USA, [8]Oklahoma City Veterans Affairs Medical Center, Oklahoma City, OK USA

To whom correspondence should be addressed: [*]Willard M. Freeman, Genes & Human Disease Program, Oklahoma Medical Research Foundation, 825 NE 13th Street, Oklahoma City, OK 73104, USA.

Tel: 405-271-3139

Fax: 405-271-2536

E-mail address: bill-freeman@omrf.org

[§]*These authors contributed equally*

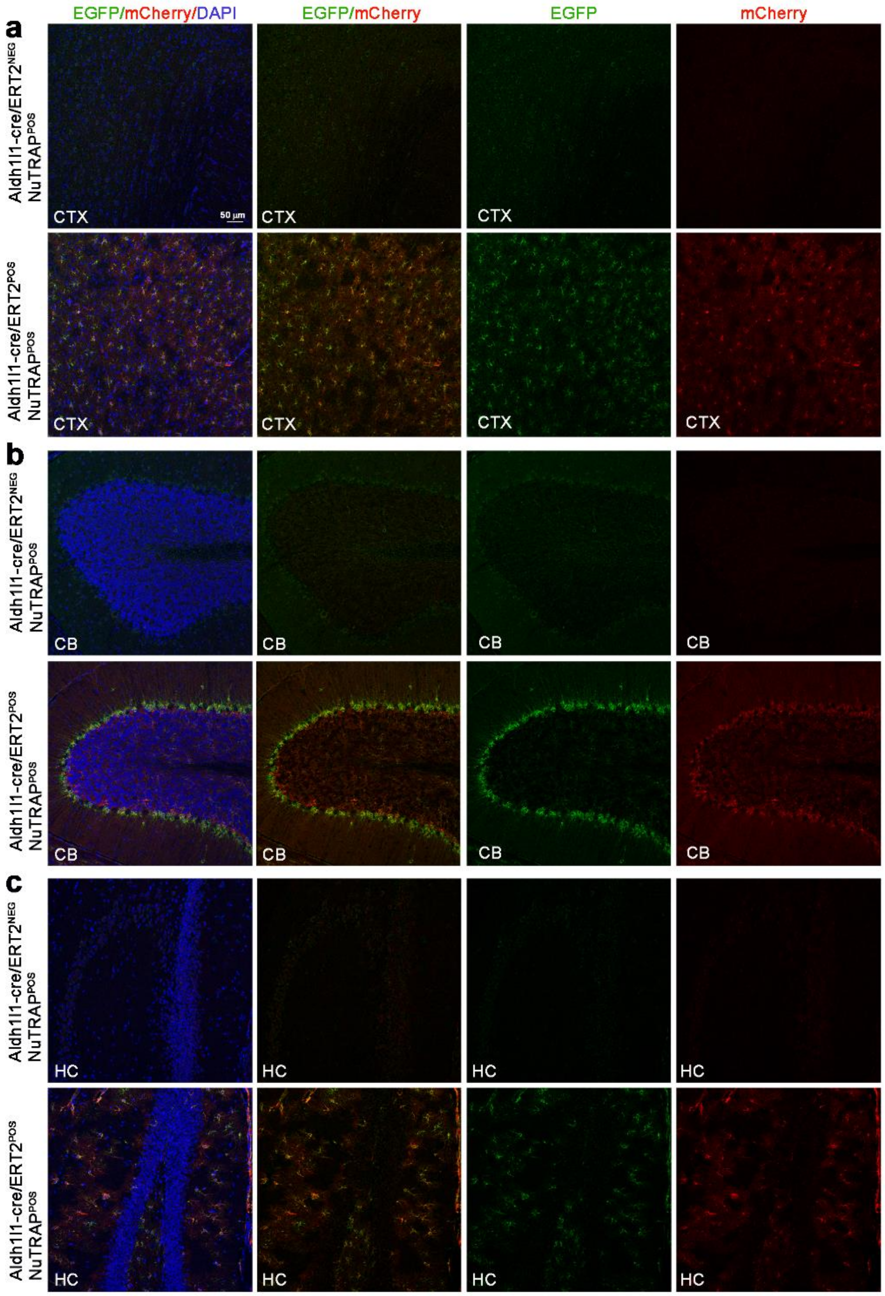

Supplementary Figure 1. DNA Recombination in brain regions of the Aldh1l1-NuTRAP model.

Aldh1l1-cre^{+} NuTRAP^{+} and cre negative NuTRAP^{+} mice were treated with Tam or left untreated and after a week brains were dissected for immunohistochemistry (IHC) analyses of frozen sections immunostained with antibodies against mCherry and GFP. Representative confocal fluorescent microscopy images of sagittal brain sections show colocalization of EGFP (green signal) and mCherry (red signal) in (**a)** cortex (CTX), (**b)** cerebellum (CB), and (**c)** hippocampus (HC) of the Aldh1l1-NuTRAP. Untreated counterparts did not display EGFP or mCherry expression. Scale bar: 50 µm.

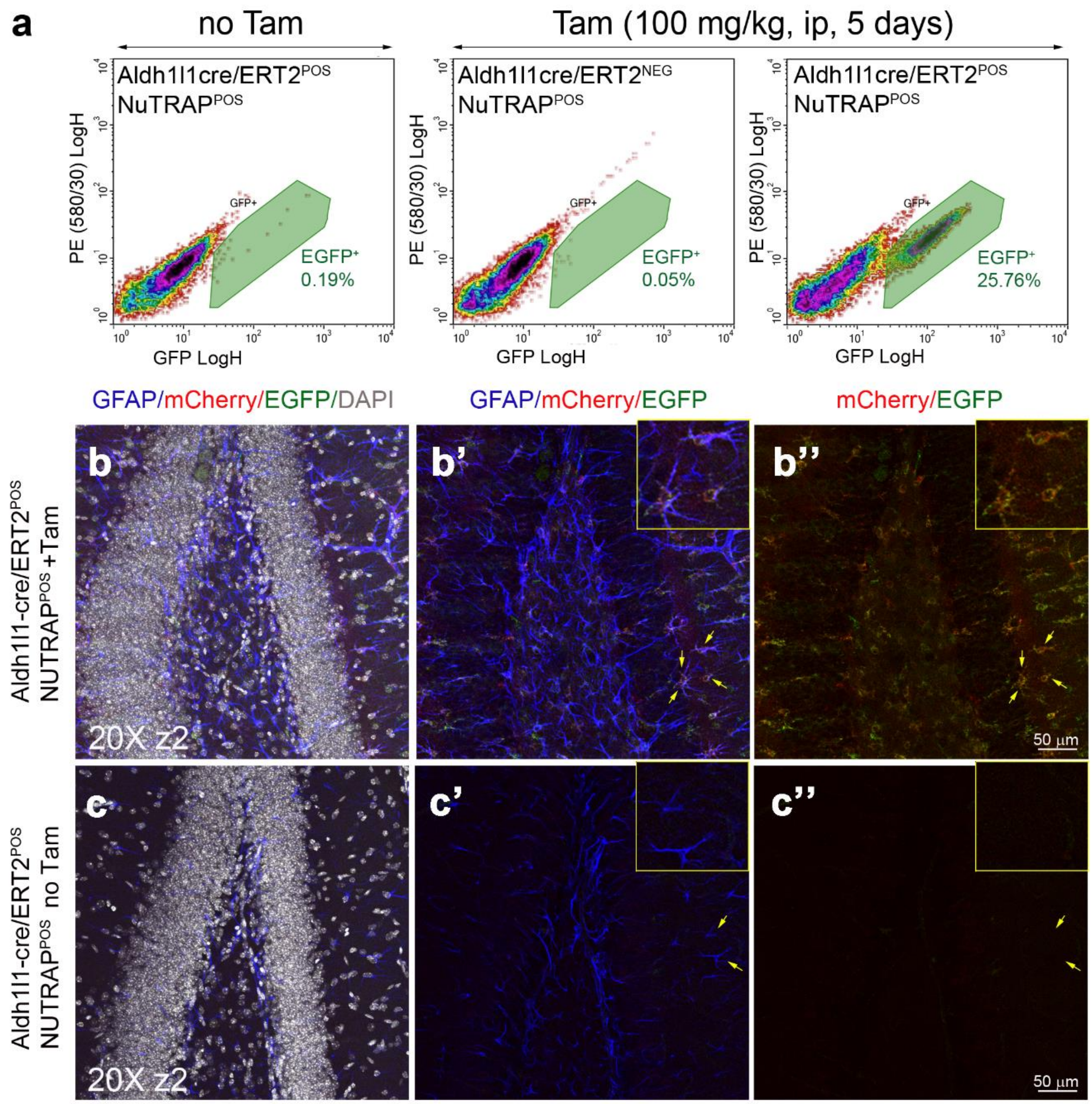

Supplementary Figure 2. Flow cytometry and immunohistochemical assessment of Tam-independent recombination in the Aldh1l1-NuTRAP brain. Aldh1l1-cre^{+} NuTRAP^{+} and cre negative NuTRAP^{+} mice were treated with Tam or left untreated and after a week brains were dissected for flow cytometry (FC) and immunohistochemistry (IHC) analyses. **a** Representative FC plots of single- cell suspensions show a distinct population of EGFP^{+} cells (25.76%) in brain samples of Aldh1l1-NuTRAP mice treated with Tam. Such EGFP^{+} cell population was negligible (0.05-0.19%) in brains of Aldh1l1-NuTRAP mice left untreated or cre negative NuTRAP positive mice treated with Tam (n=2/group). **b-c''** Representative confocal fluorescent microscopy images of sagittal brain sections captured in the dentate gyrus of the hippocampus show colocalization of EGFP (green signal) and mCherry (red signal) to GFAP expressing cells (blue signal: astrocytes) in the Aldh1l1-NuTRAP. Untreated counterparts did not display EGFP or mCherry expression. In panel b'-b'' and c'-c'', cells depicted with yellow arrows are shown in the insets (2X digital zoom) (n=3/group). Scale bar: 50μm.

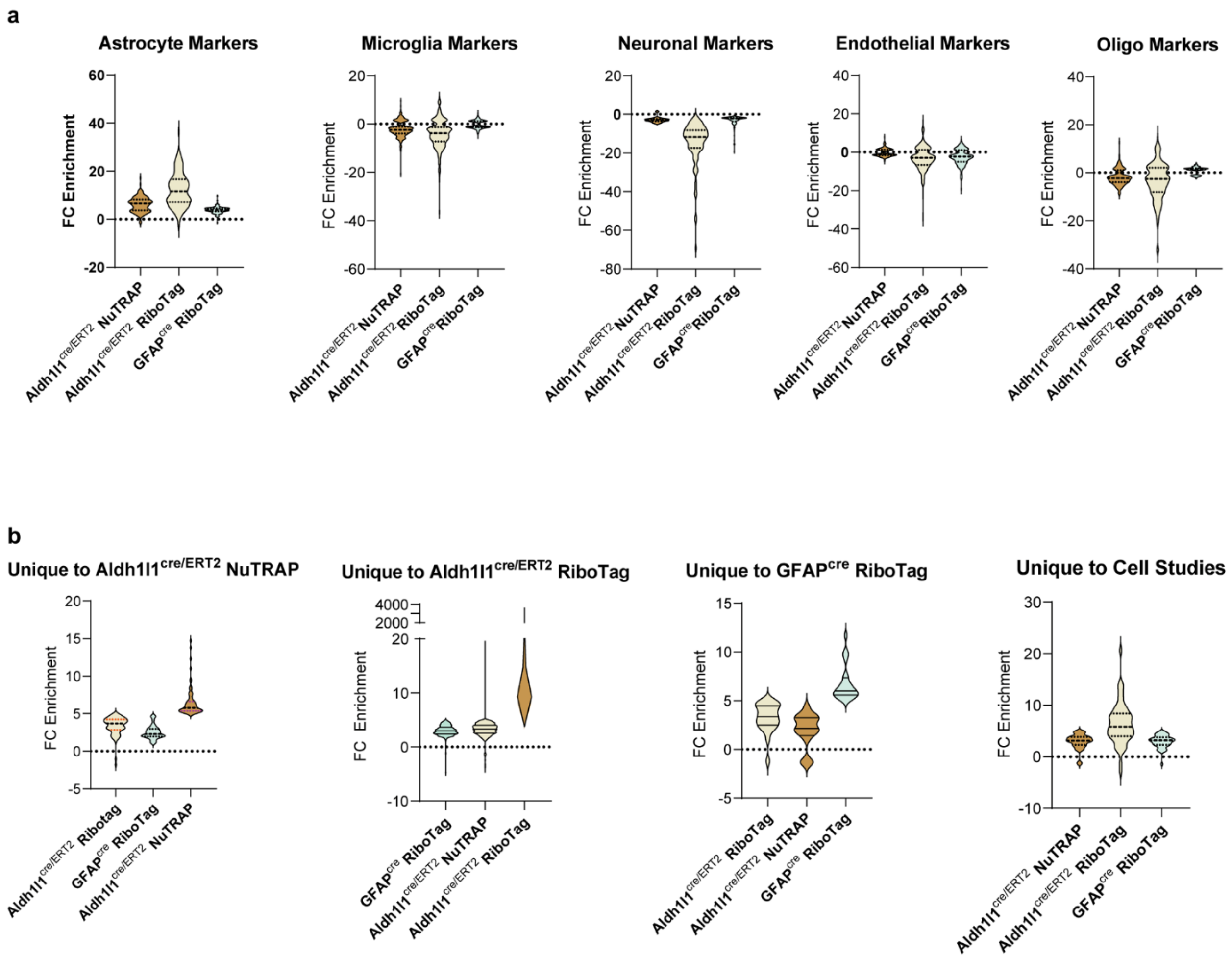

Supplementary Figure 3: Enrichment distributions. a Distributions of enrichments in gene expression (Positive fraction/Input) for each of the ribosomal profiling methods against the cell type marker gene lists developed from cell sorting studies (Supplementary Data 1). **b** Distributions of enrichments for genes observed as markers (statistically significant, fold change >5) in only one ribosomal profiling study (Figure 3e) or from the cell sorting studies (Figure 3g).

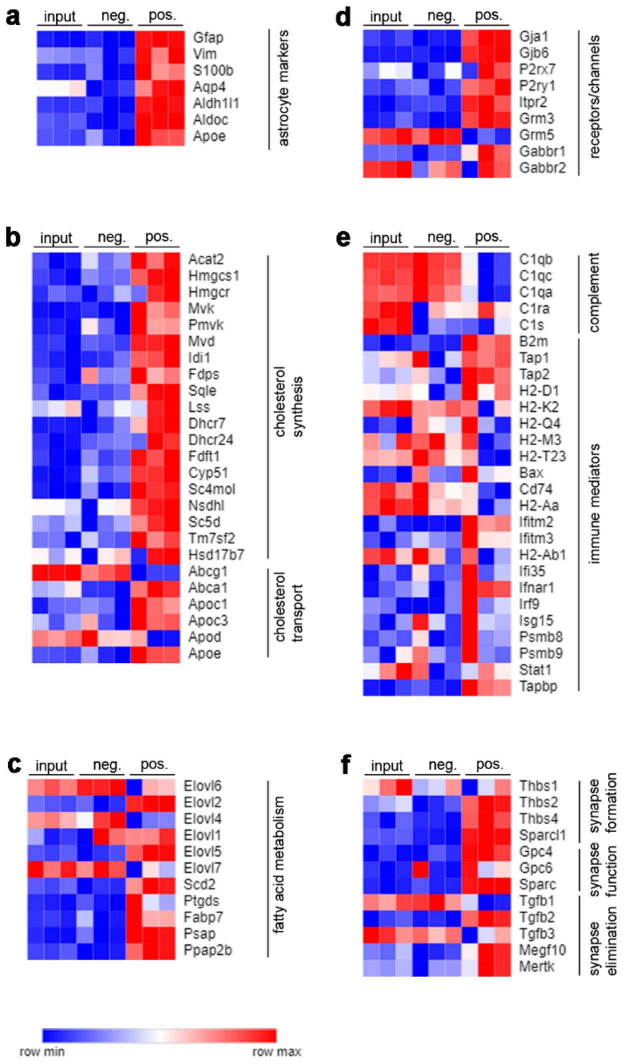

Supplementary Figure 4. Differential gene expressions in Aldh1l1-NuTRAP positive fraction strongly correlate with physiological functions of astrocytes in the brain. Heatmaps show in the TRAP positive fraction of Aldh1l1-NuTRAP brains is: (**a**) enriched in astrocyte marker genes compared to input, in agreement with over-representation of genes that are critical in astrocyte physiological functions such as **(b)** cholesterol synthesis and transport, **(c)** fatty acid metabolism, **(d)** receptors/channels, and **(f)** synapse modification (formation, function, and elimination) processes while **(e)** under-representation of complement/immune mediators, commonly associated with microglial function.

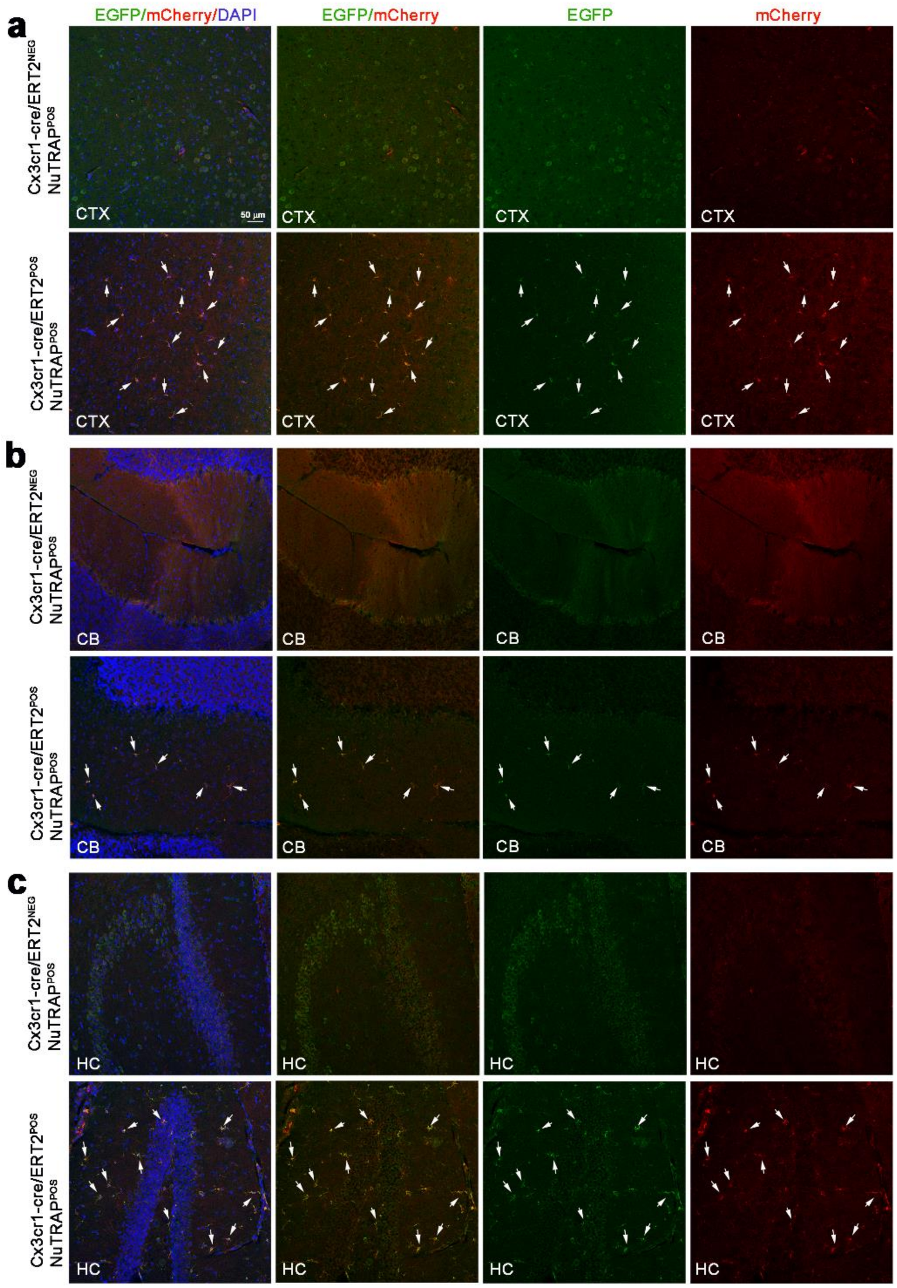

Supplementary Figure 5. DNA Recombination in brain regions of the Cx3cr1-NuTRAP model. Cx3cr1-cre$^+$ NuTRAP$^+$ and cre negative NuTRAP$^+$ mice were treated with Tam or left untreated and after 4 weeks brains were dissected for immunohistochemistry (IHC) analyses of frozen sections immunostained with antibodies against mCherry and GFP. Representative confocal fluorescent microscopy images of sagittal brain sections show colocalization of EGFP (green signal) and mCherry (red signal) in **(a)** cortex (CTX), **(b)** cerebellum (CB), and **(c)** hippocampus (HC) of the Cx3cr1-NuTRAP. Untreated counterparts did not display EGFP or mCherry expression, but not specific background (noted especially in CTX). Scale bar: 50 µm.

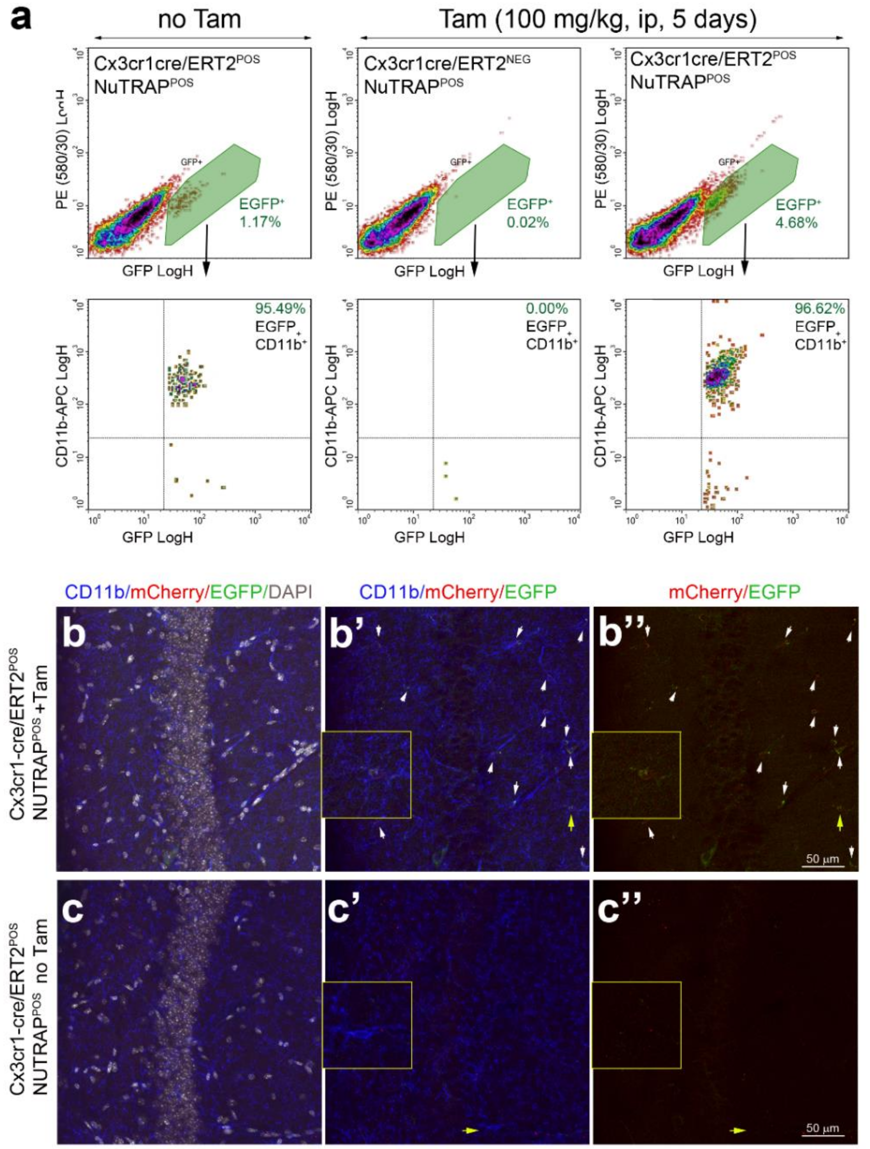

Supplementary Figure 6. Flow cytometry and immunohistochemical assessment of Tam-independent recombination in the Cx3cr1cre-NuTRAP brain. Cx3cr1-cre/ERT2$^+$; NuTRAP$^+$ and Cx3cr1-cre/ERT2$^-$; NuTRAP$^+$ mice were treated with Tam or left untreated and after three weeks brains were dissected for flow cytometry (FC) and immunohistochemistry (IHC) purposes. **a** Representative FC plots of single- cell suspensions show a distinct population of EGFP$^+$ cells (4.68%) in brain samples of Cx3cr1-cre/ERT2$^+$ mice treated with Tam that almost exclusively co-expressed CD11b (96.62%) and was undetectable (0.02 %) in brains of Cx3cr1-cre/ERT2$^-$ held to the same treatment. In Cx3cr1-cre/ERT2$^+$ mice that did not receive Tam, EGFP+ were detected at a lesser level than in treated counterparts (1.17%) (n=2/group). **b-c''** Representative confocal fluorescent microscopy images of sagittal brain sections captured in the hippocampus show colocalization of EGFP (green signal) and mCherry (red signal) to CD11b expressing cells (blue signal) in the Aldh1l1-cre/ERT2$^+$. Untreated counterparts had almost no EGFP or mCherry expression. In panel b'-b'' and c'-c'', cells depicted with yellow arrows are shown in the insets (2X digital zoom) (n=3/group). Scale bar: 50 µm.

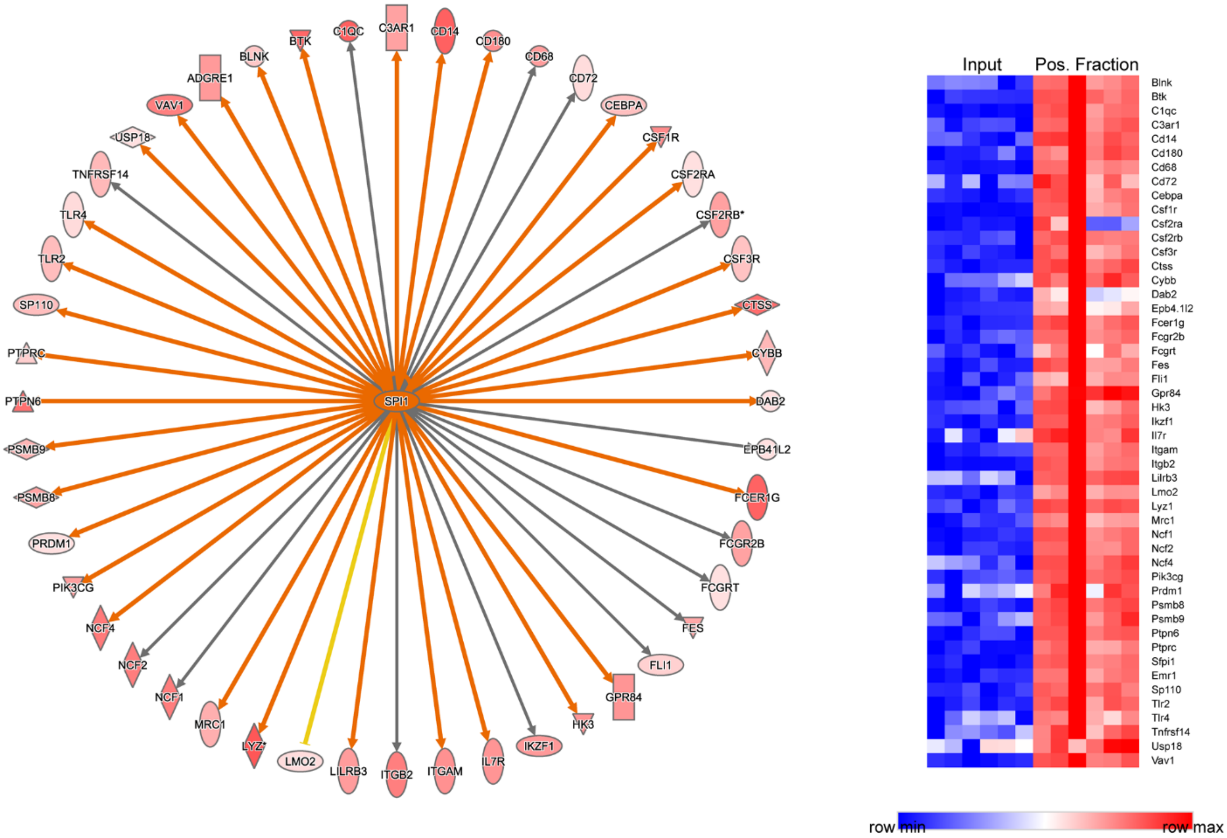

Supplementary Figure 7. Differential gene expressions in Cx3cr1-NuTRAP positive fraction correlate with enrichment of canonical targets of microglial SPI1 in the brain. Genes enriched in the microglia transcriptome included an overrepresentation of genes regulated by PU.1 (also known as Spi1), a transcription factor that shapes the homeostatic functions of microglia.

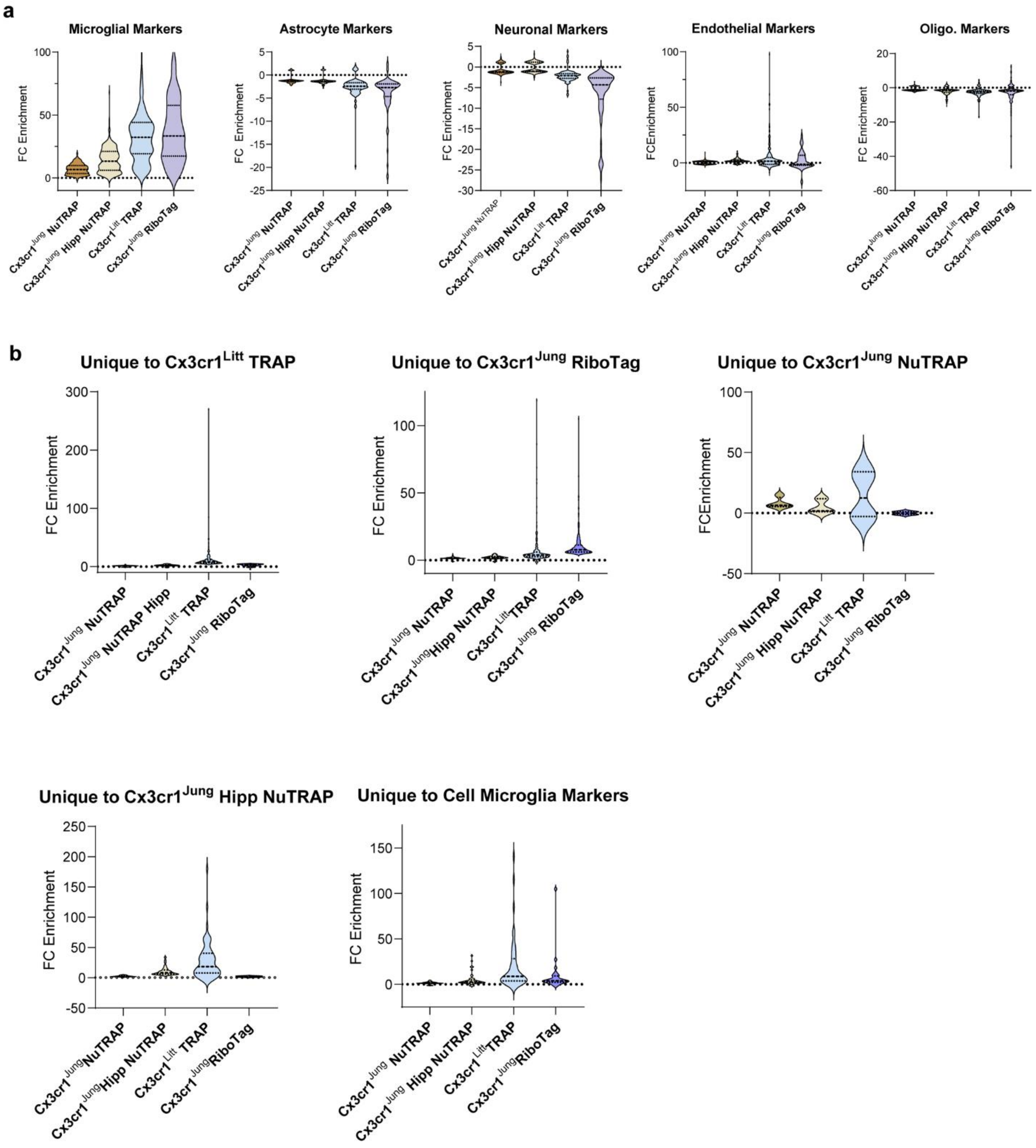

Supplementary Figure 8. Enrichment distributions. a Distributions of enrichments in gene expression (Positive fraction/Input) for each of the ribosomal profiling methods against the cell type marker gene lists developed from cell sorting studies (Supplementary Data 2). **b** Distributions of enrichments for genes observed as markers (statistically significant, fold change >5) in only one ribosomal profiling study (Figure 6e) or from the cell sorting studies (Figure 7g).

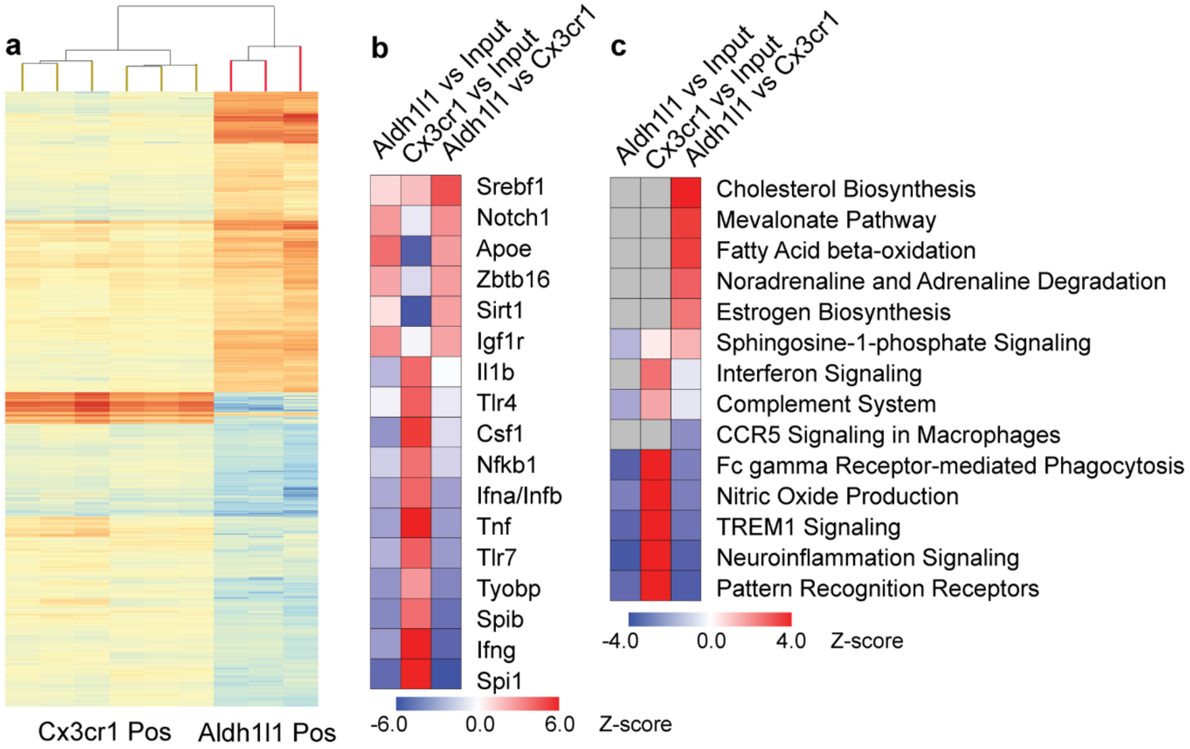

Supplementary Figure 9. Transcriptomic comparison of astrocytes and microglia. a Positive fractions from Aldh1l1-NuTRAP and Cx3cr1-NuTRAP were compared. **b** Regulator analysis comparison. **c** Pathway analysis comparison.

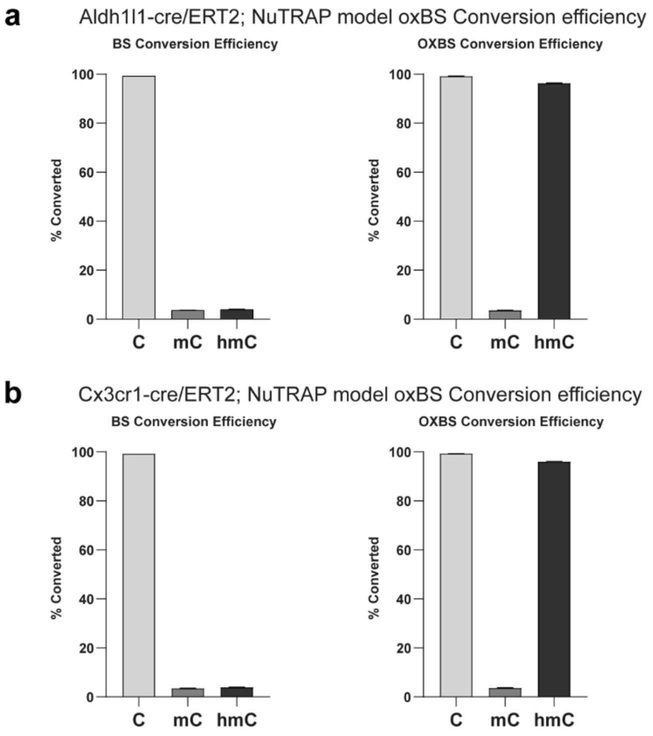

Supplementary Figure 10. Conversion efficiency of CEGX spike-in controls. Exogenous control sequences (CEGX, Cambridge, UK) with methylation and hydroxymethylation at specific bases were spiked in to each sheared DNA sample (0.04% w/w) prior to oxidation and/or bisulfite conversion. After sequencing, raw fastq files were run through CEGXQC v0.2, a custom FASTQC program, to generate summary documents and QC reports based on the conversion performance of the spike-in sequencing controls. **a-b** Conversion percentages for different cytosine modifications (C, mC, and hmC) are plotted for bisulfite-converted and oxidative bisulfite-converted libraries. Bisulfite-converted libraries show high conversion of unmodified cytosines and low conversion of methylated and hydroxymethylated cytosines. While oxidative bisulfite-converted libraries show high conversion of unmodified cytosines and hydroxymethylated cytosines, and low conversion of methylated cytosines. The bisulfite-converted libraries are used to determine the total percent modified cytosines (mC+hmC), while the oxidative bisulfite-converted libraries are used to determine the percent methylated cytosines (mC).

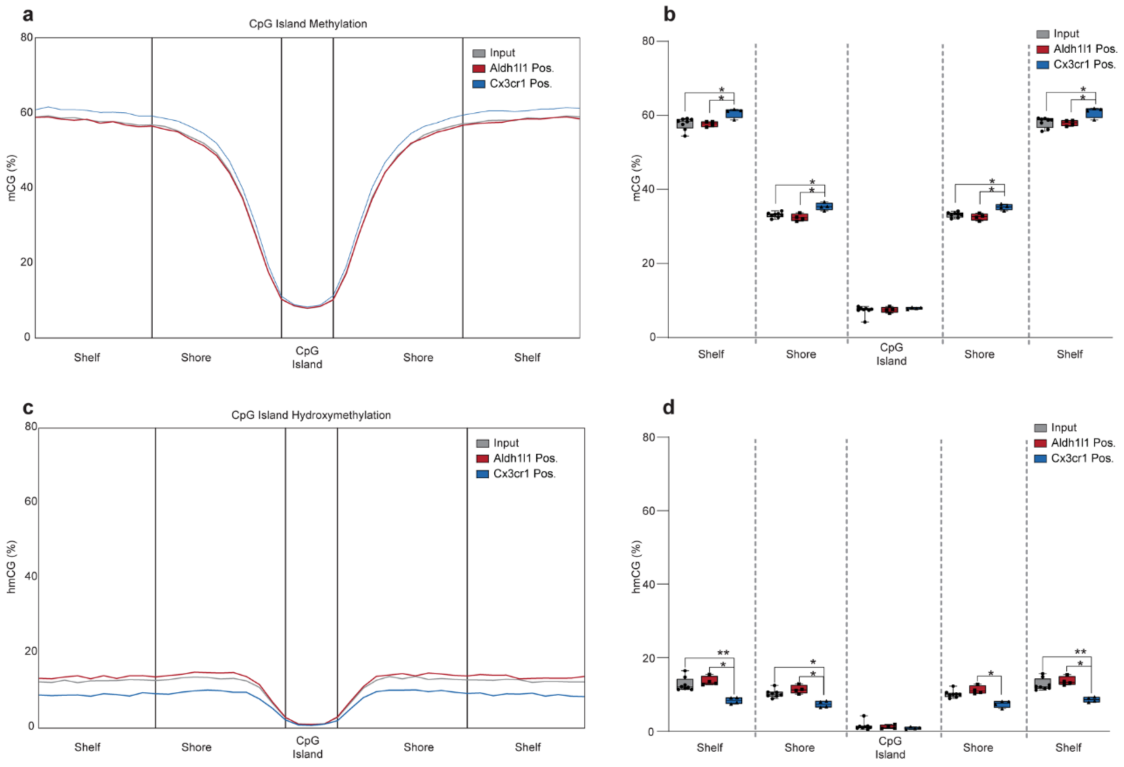

Supplementary Figure 11. CpG island, shore and shelf methylation and hydroxymethylation in the Aldh1l1- NuTRAP and Cx3cr1- NuTRAP mouse brains by WGoxBS. After Tam treatment, half brain hemispheres were harvested from Aldh1l1-NuTRAP and Cx3cr1-NuTRAP mice and subjected to nuclei isolation and subsequent INTACT protocol for genomic DNA extraction for epigenome analyses. Analysis of methylation and hydroxymethylation levels covering CpG islands, shores, and shelves revealed that the shores and shelves of Cx3cr1-NuTRAP INTACT positive fractions cells had significantly higher mCG levels **(a-b)** and significantly lower hmCG levels **(c-d)** compared to the other groups. (n=8/input group, n=4/positive fraction group; 2-way ANOVA with Tukey's multiple comparison test, *p<0.05, **p<0.01).

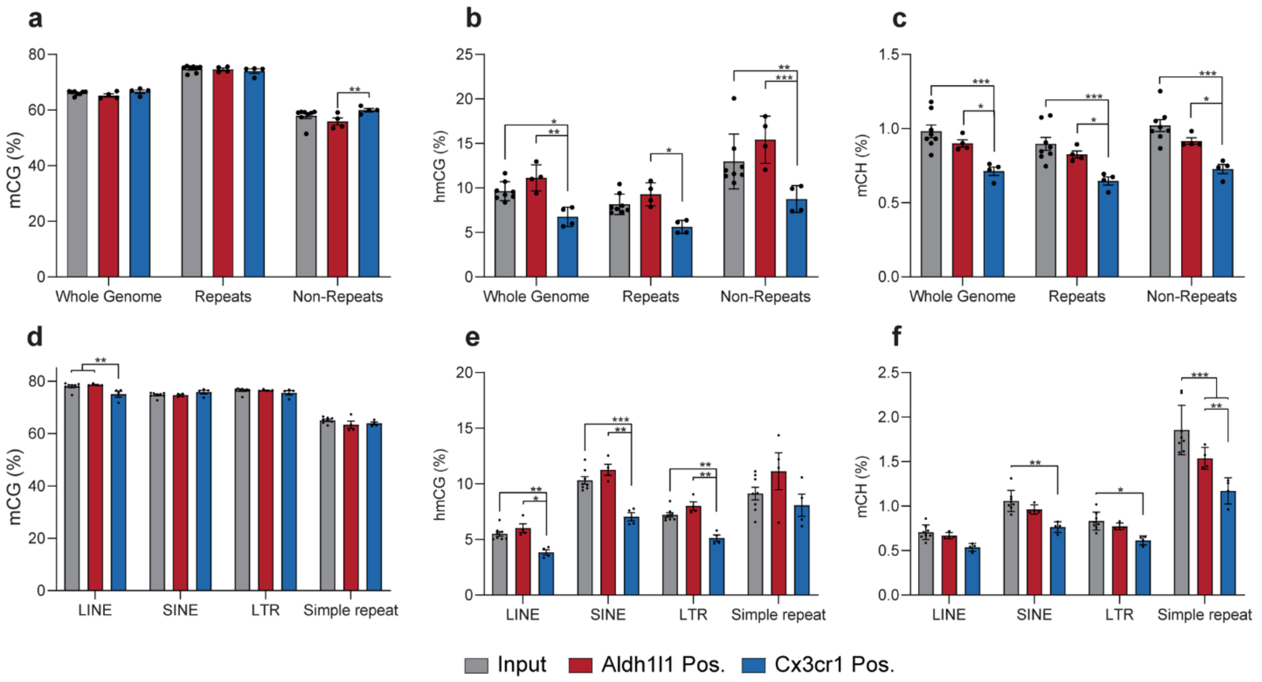

Supplementary Figure 12. Comparison of whole genome, repeat, and non-repeat levels of methylation and hydroxymethylation between input and positive fractions of INTACT-isolated DNA from Aldh1l1-NuTRAP and Cx3cr1-NuTRAP mouse brains. a Overall repetitive elements have higher levels of CG methylation and non-repetitive elements have lower levels of CG methylation than whole genome levels. While there are no differences in total mCG between the input DNA, Aldh1l1+ DNA, and Cx3cr1+ DNA in the whole genome or repeat elements, there is a small, but significant, difference in mCG between Aldh1l1^{+} DNA and Cx3cr1^{+} DNA in non-repeat elements of the genome. **b** There is less hmCG of Cx3cr1+ DNA than both input DNA and Aldh1l1+ DNA at the whole genome level and in repetitive and non-repetitive elements. **c** There is less mCH of Cx3cr1+ DNA than both input DNA and Aldh1l1+ DNA at the whole genome level and in repetitive and non-repetitive elements. **d** LINEs contain less mCG in Cx3cr1+ DNA than in input DNA or Aldh1l1+ DNA. **e** LINEs, SINEs, and LTRs have lower hmCG levels in Cx3cr1+ DNA than in input DNA or Aldh1l1+ DNA**. f** SINEs and LTRs have lower mCH levels in Cx3cr1+ DNA than input DNA. Simple repeats have lower mCH levels in Cx3cr1+ DNA than in input DNA and Aldh1l1+ DNA. Aldh1l1+ DNA has lower mCH levels in simple repeats than input DNA. (n=8/input group, n=4/positive fraction group; 2-way ANOVA with Tukey's multiple comparison's test, *p<0.05, **p<0.01, ***p<0.001).

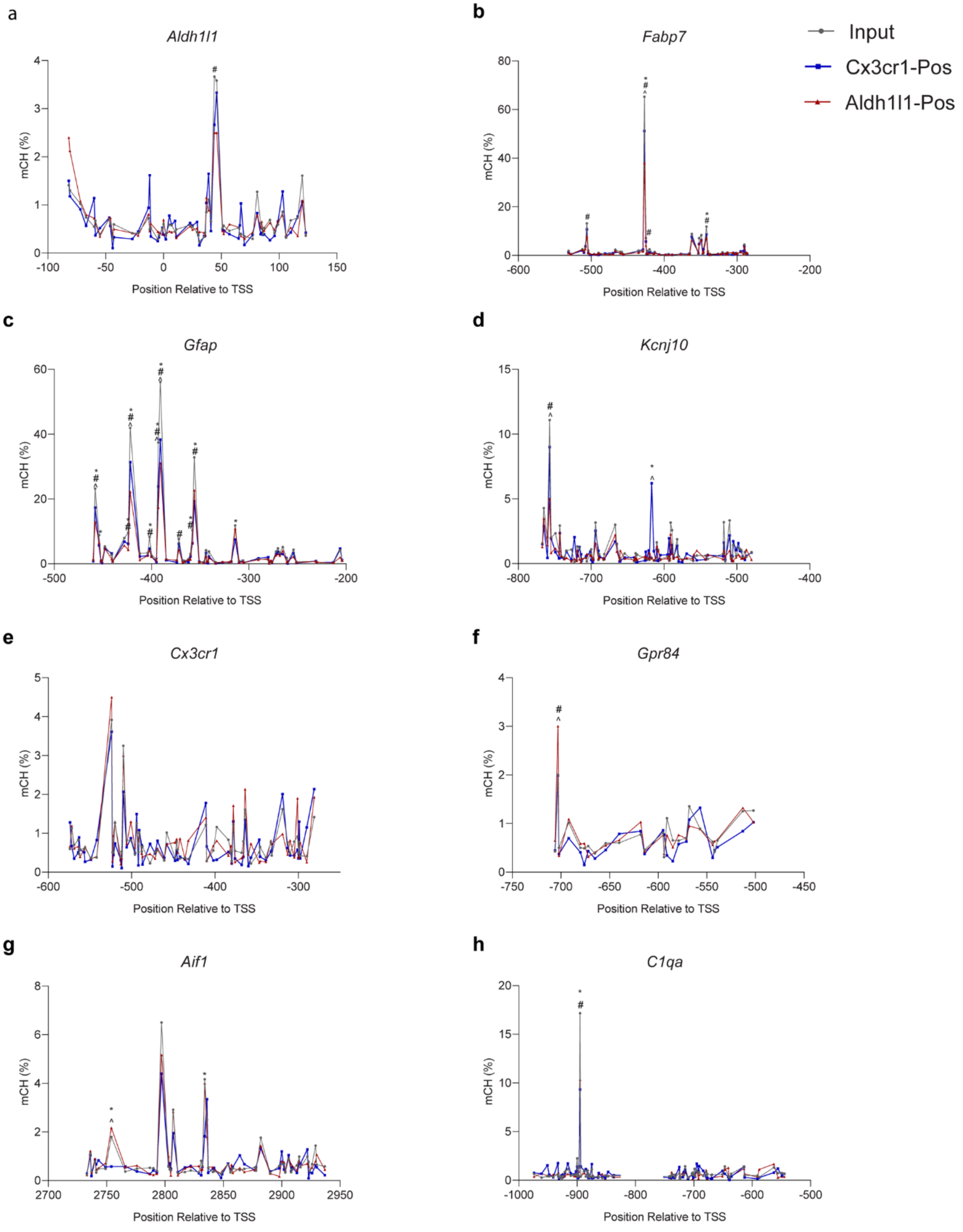

Supplementary Figure 13. Non-CpG methylation (mCH) in specific gene promoters and intragenic regions in Cx3cr1-NuTRAP and Aldh1l1-NuTRAP mouse brains by targeted

BSAS. DNA from input, Cx3cr1-NuTRAP positive fraction (Cx3cr1-Pos), and Aldh1l1-NuTRAP positive fraction (Aldh1l1-Pos) was assayed for region-specific mCH in astrocytic (*Aldh1l1, Fabp7, Gfap, Kcnj10*) and microglial (*Cx3cr1, C1qa, Aif1, Gpr84*) cell marker genes by targeted BSAS for the same regions as in Figure 10. Average CH methylation (% mCH) at each CH site within the displayed amplicon is plotted. Sites with greater than 1% differences in mCH between at least two groups were analyzed for differential methylation (n=6/group; Two-way ANOVA with Tukey's post-hoc; *$p<0.05$ Input v. Cx3cr1-Pos, #$p<0.05$ Input v. Aldh1l1, ^$p<0.05$ Cx3cr1-Pos v. Aldh1l1-Pos). **a-d** For each astrocytic marker gene region assessed (*Aldh1l1, Fabp7, Gfap, Kcnj10*) there was at least one CH site that was differentially methylated between input, Aldh1l1-Pos, and/or Cx3cr1-Pos fractions. **e** There were no site-specific differences in mCH within the region of *Cx3cr1* between input, Cx3cr1-Pos, and Aldh1l1-Pos fractions. **f-h** For each microglial marker gene region assessed (*C1qa, Aif1, Gpr84*) there was at least one CH site that was differentially methylated between input, Aldh1l1-Pos, and/or Cx3cr1-Pos fractions.

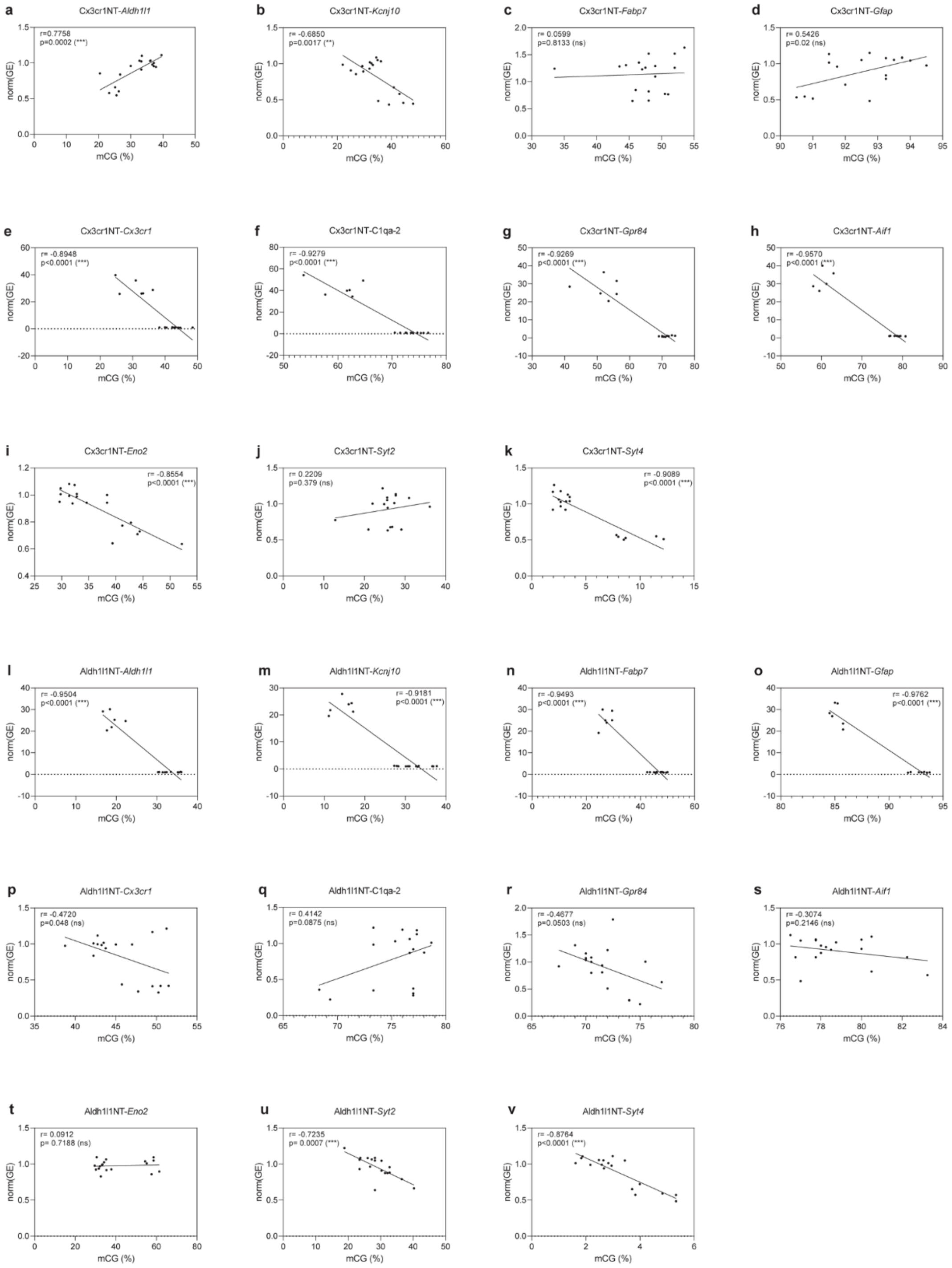

Supplementary Figure 14. Correlation between gene promoter methylation and expression in Cx3cr1-NuTRAP and Aldh1l1-NuTRAP brains. a-k DNA and RNA were isolated from

Cx3cr1-NuTRAP brains (input, negative, and positive fractions) for paired targeted BSAS and qPCR. Correlation of average CG methylation (% mCG) with gene expression (normalized RQ) shows strong negative correlations (Pearson's r; Bonferonni correction for multiple comparisons; *$p<0.0045$) within microglial-specific marker genes (*Cx3cr1, C1qa, Gpr84, Aif1*). **I-v** DNA and RNA were isolated from Aldh1l1-NuTRAP brains (input, negative, and positive fractions) for paired targeted BSAS and qPCR. Correlation of average CG methylation (% mCG) with gene expression (normalized RQ) shows strong negative correlations (Pearson's r; Bonferonni correction for multiple comparisons; *$p<0.0045$) within astrocyte-specific marker genes (*Aldh1l1, Kcnj10, Fabp7, Gfap*).

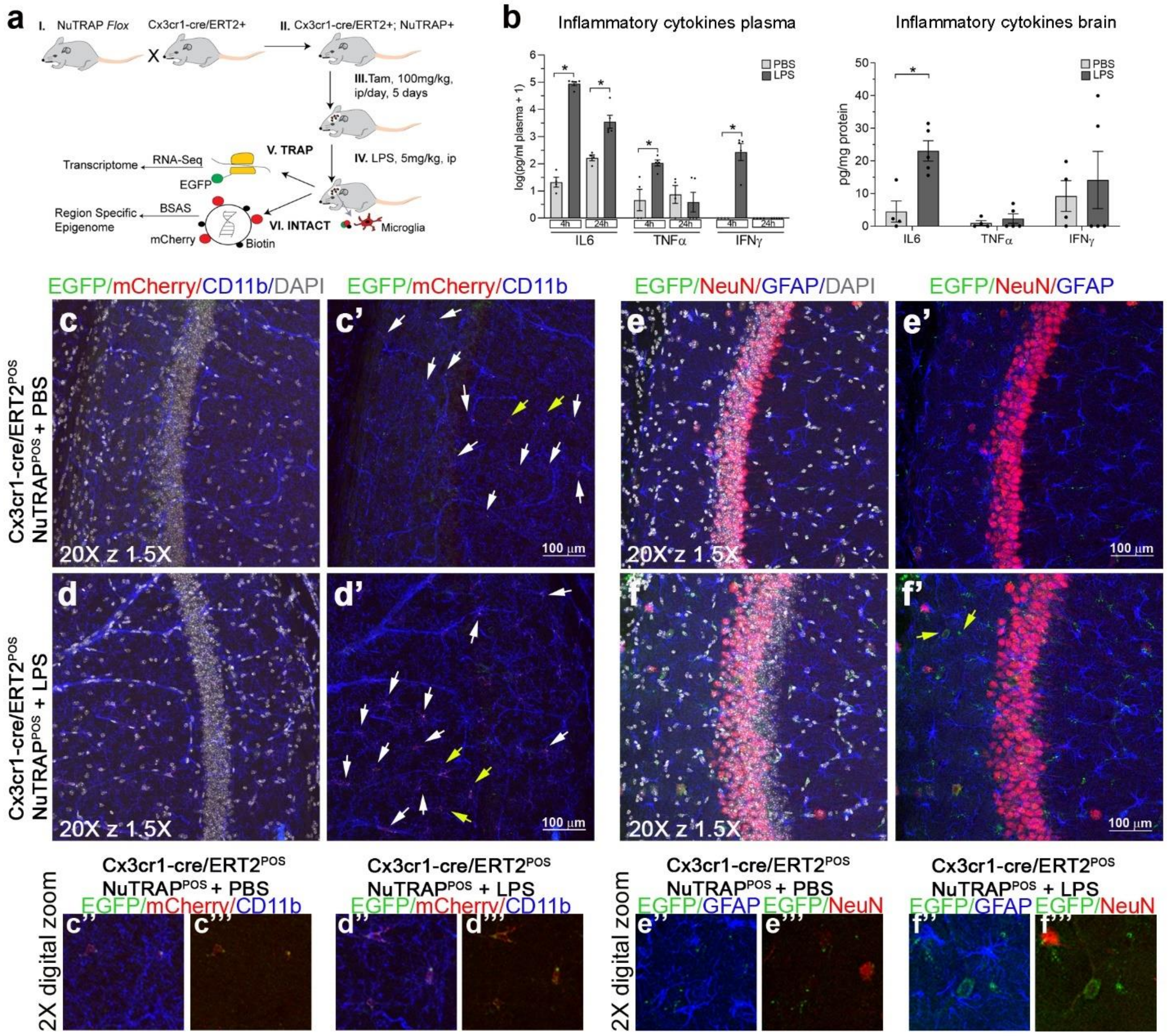

Supplementary Figure 15. LPS systemic treatment as experimental demonstration of the Cx3cr1-cre/ERT2 model. a Schematic of the experimental design for epigenetic and transcriptomic analyses of brain microglia upon LPS challenge. 3-4 weeks after Tam treatment, Cx3cr1-cre/ERT2$^+$; NuTRAP$^+$ mice were subjected to a single ip injection with LPS or PBS as control and 24 h later their brains dissected for protein and IHC purposes. **b** Validation of systemic LPS treatment. Blood samples were collected at 4 and 24h after LPS injection before euthanasia and brain harvest. Plasma samples and brain tissue homogenates were used to measure concentration of inflammatory cytokines IL-6, TNFα, and IFNɣ by suspension array. Values are expressed as average pg analyte/ml ± SEM in plasma (4h and 24h time points) and average pg analyte/mg ±SEM in tissue (24h time point). **c-f'** Representative confocal fluorescent microscopy images of sagittal brain sections captured in the hippocampus show EGFP expression (green signal) was found in cells that co-expressed mCherry (red signal) and CD11b (blue signal). **c''-f'''** 2X digital zoom on cells depicted with yellow arrows in **(c'-d')** and **(f')** show co-localization of EGFP and mCherry with CD11b but not with GFAP or NeuN. * $p<0.05$ between PBS and LPS treated groups for each analyte by unpaired T test (n=4/PBS group and 5/LPS group). Scale bar: 100 µm.

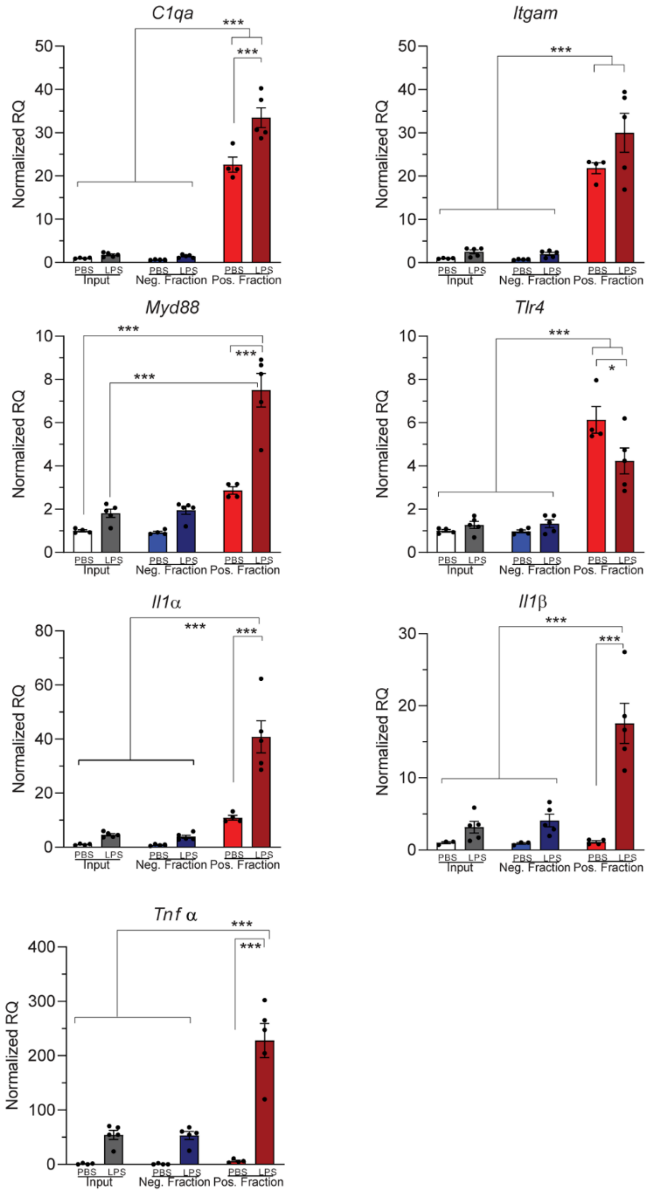

Supplementary Figure 16. qPCR validation of TRAP-RNA in the Cx3cr1-cre/ERT2 model following LPS treatment. 3-4 weeks after Tam treatment, Cx3cr1-cre/ERT2^{+}; NuTRAP^{+} mice were subjected to a single ip injection with LPS or PBS as control and 24 h later their brains harvested and one hemisected half used for TRAP isolation of RNA and downstream analyses. qPCR analysis of microglial genes (*C1qa* and *Itgam*) and candidate genes related to LPS-induced inflammation (*Tlr4, Myd88, Il1α, Il1β,* and *Tnfα*) demonstrate both enrichment and higher magnitude changes in the positive fraction as compared to the input. Bar graphs represent average RQ ± SEM for each gene expression measured. *, *** p<0.05 and p<0.001 respectively by two-way ANOVA followed by the Sidak's multiple comparison test (n=4 for PBS group and n=5 for LPS group).

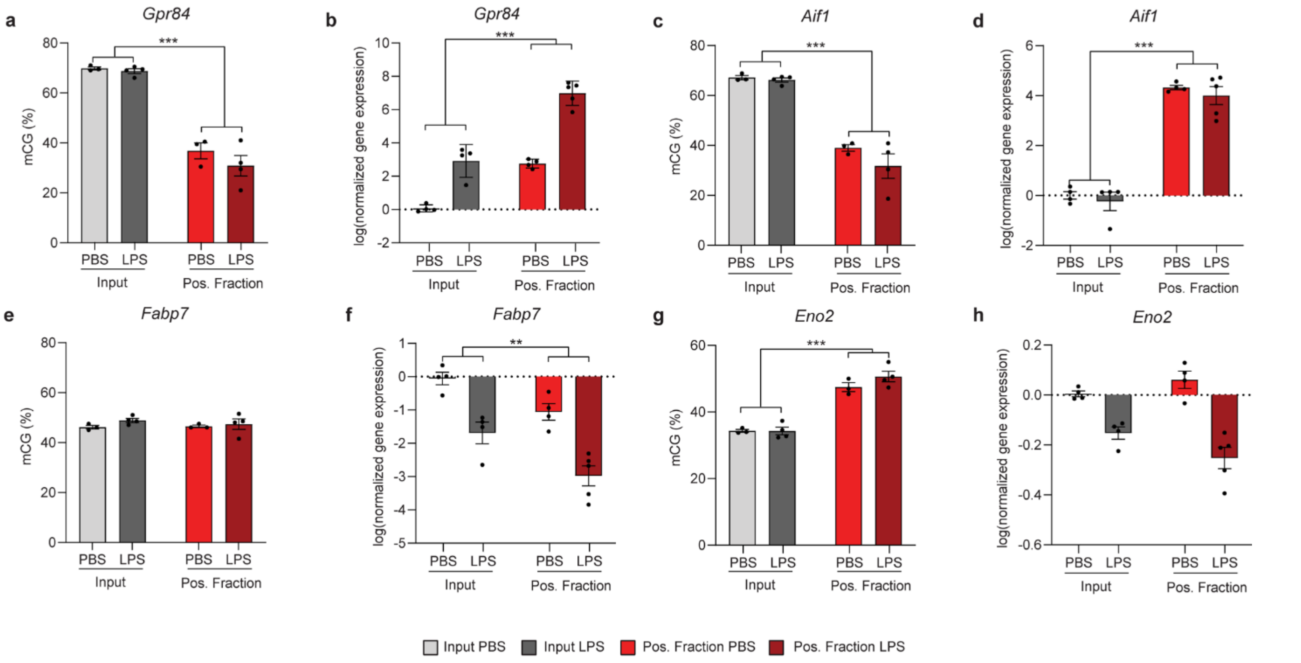

Supplementary Figure 17. Targeted bisulfite amplicon sequencing (BSAS) to assess methylation (mCG) of microglial DNA in cell-specific marker gene promoters 24 hours after LPS challenge in Cx3cr1-NuTRAP mouse brain. *Gpr84*, a microglial marker gene, has (**a**) lower CG promoter methylation, by BSAS, and (**b**) higher gene expression, by RNA-Seq, in the INTACT-isolated positive fraction than the input, regardless of LPS treatment. *Aif1*, a microglial marker gene, has (**c**) lower CG promoter methylation, by BSAS, and (**d**) higher gene expression, by RNA-Seq, in the INTACT-isolated positive fraction than the input, regardless of LPS treatment. *Fabp7*, an astrocyte marker gene, has (**e**) no difference in promoter CG methylation between input and INTACT-isolated positive fraction, by BSAS, and (**f**) higher gene expression, by RNA-Seq, in the INTACT-isolated positive fraction than the input, regardless of LPS treatment. (**g,h**) *Eno2*, a neuronal marker gene, has higher CG promoter methylation by BSAS (**g**), and no difference in gene expression by RNA-Seq (**h**), in the INTACT-isolated positive fraction than the input, regardless of LPS treatment. (2-way ANOVA or mixed effects analysis with Holm-Sidak correction for multiple comparisons, main effect *p<0.05, **p<0.01, and ***p<0.001).

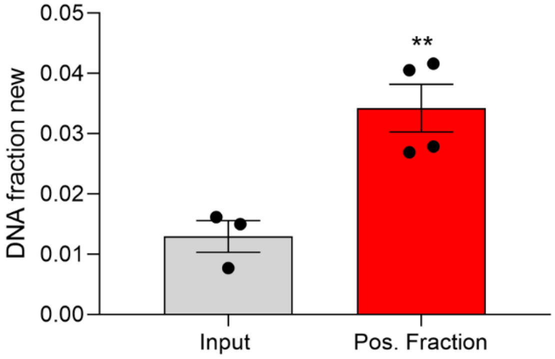

Supplementary Figure 18. Stable isotope labeling study in the Cx3cr1-NuTRAP mouse brain. Microglial proliferation was measured as incorporation of deuterium into purine deoxyribose. Mice were given an intraperitoneal injection of 99.9% D_2O and subsequently provided drinking water enriched with 8% D_2O for 30 days. Following INTACT-DNA isolation, DNA was hydrolyzed for analysis of the pentafluorobenzyl-*N*,N-di(pentafluorobenzyl) derivative of deoxyribose by GC-MS. Fraction of new DNA was calculated based on the product/precursor relationship in samples from input and positive INTACT fractions. Bar graphs represent average DNA fraction new ± SEM, ** $p<0.01$ by two-tailed unpaired T test comparison (n=3/input and n=4/positive fraction).

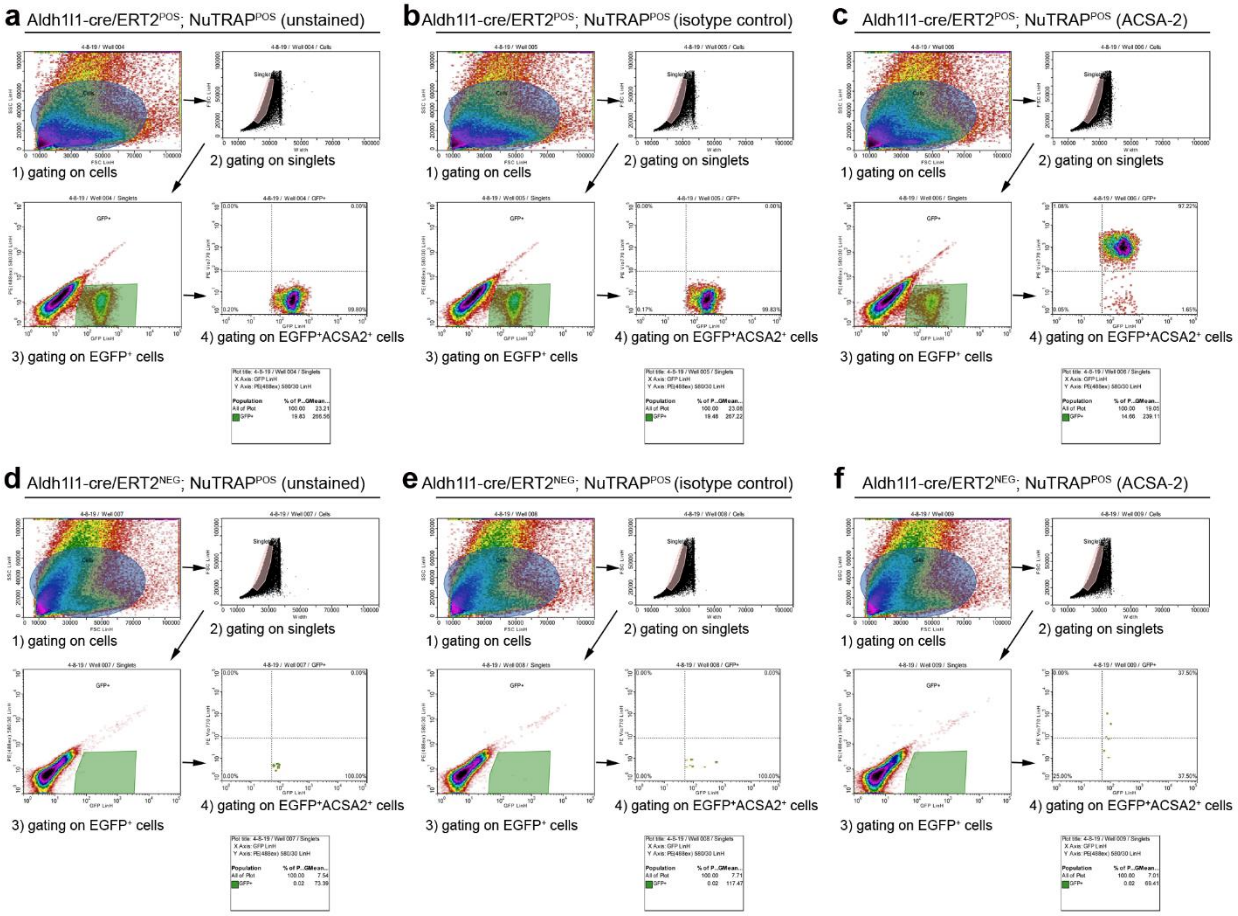

Supplementary Figure 19. Gating strategy for flow cytometry analysis of tamoxifen induced cre recombination in the Aldh1l1-NuTRAP model. Representative density plots from flow cytometry results illustrate steps followed for gating on EGFP^{+}ACSA2^{+} cells (astrocytes) from single-cell suspension of Aldh1l1-cre/ERT2^{+}NuTRAP^{+} brains analyzed. The gating process described under Methods section is applied to **(a)** unstained, **(b)** isotype control-PE-Vio770, and **(c)** ACSA-2-PE-Vio770 stained cells from the same brain cell suspension. 1) Cells are gated in the scatter range and for (2) subsequent selection of single cells (singlets). 3) Using a combination of filters, singlets are further gated for positive selection of EGFP^{+} cells that are subsequently selected as ACSA2^{+} (4: astrocytes). Same gating strategy applied to cre negative counterparts is represented in **(d-e-f).** Note: Samples in c and f are the same used to show representative data in Figure 2.

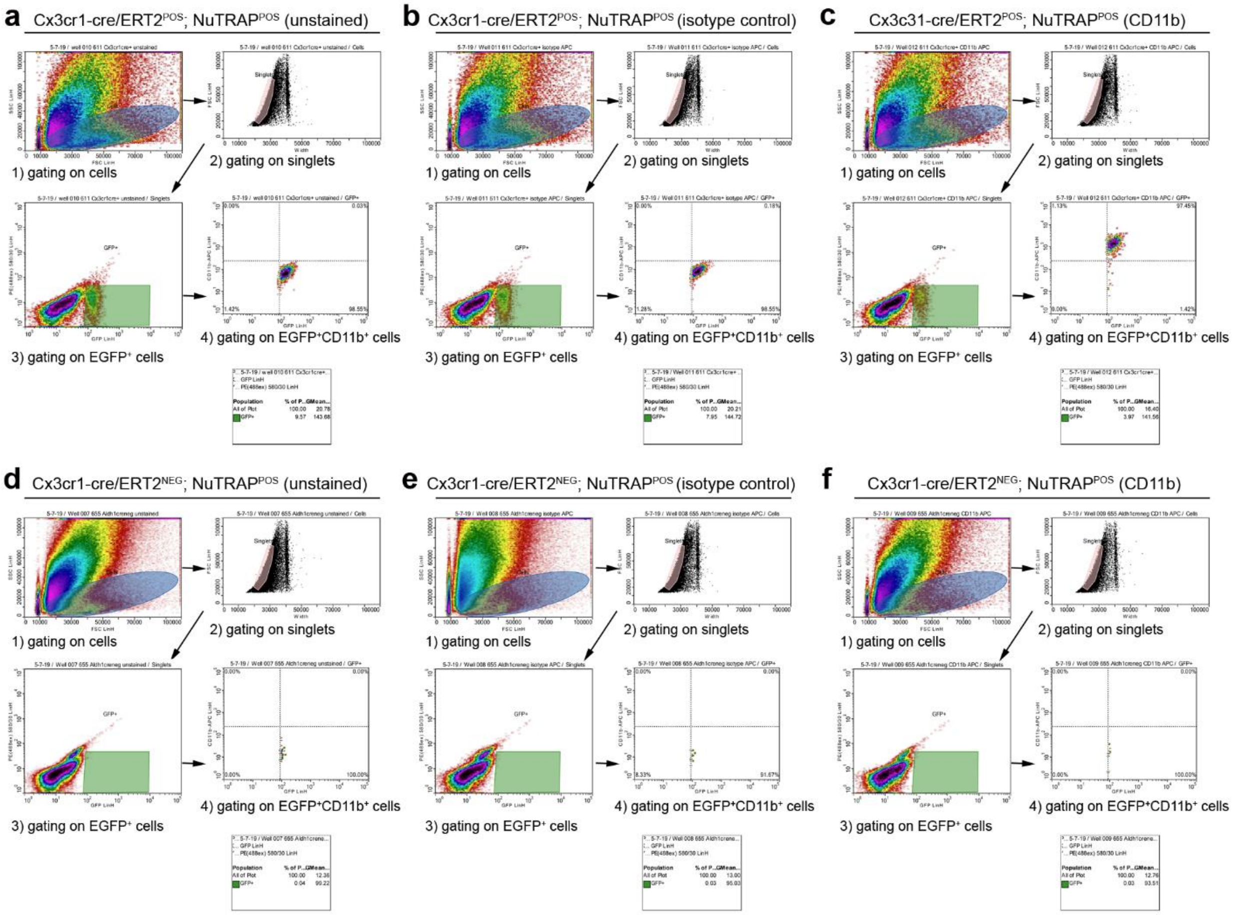

Supplementary Figure 20. Gating strategy for flow cytometry analysis of microglia in the Cx3cr1-NuTRAP model. Representative density plots from the flow cytometry results illustrate steps followed for gating on EGFP$^+$CD11b$^+$ cells (microglia) from single-cell suspension of Cx3cr1-NuTRAP brains analyzed. The gating process described under Methods section is applied to **(a)** unstained, **(b)** isotype control-APC, and **(c)** CD11b-APC stained cells from the same brain cell suspension. 1) Cells are gated in the scatter range for (2) subsequent selection of single cells (singlets). 3) Using a combination of filters, singlets are further gated for positive selection of EGFP$^+$ cells that are subsequently selected as CD11b$^+$ (4: microglia). Same gating strategy applied to cre negative sample (Aldh1l1cre/ERT2$^-$; NuTRAP$^+$) is represented in **(d-e-f)**. Note: samples in C and F are the same used to show representative data in Figure 5.

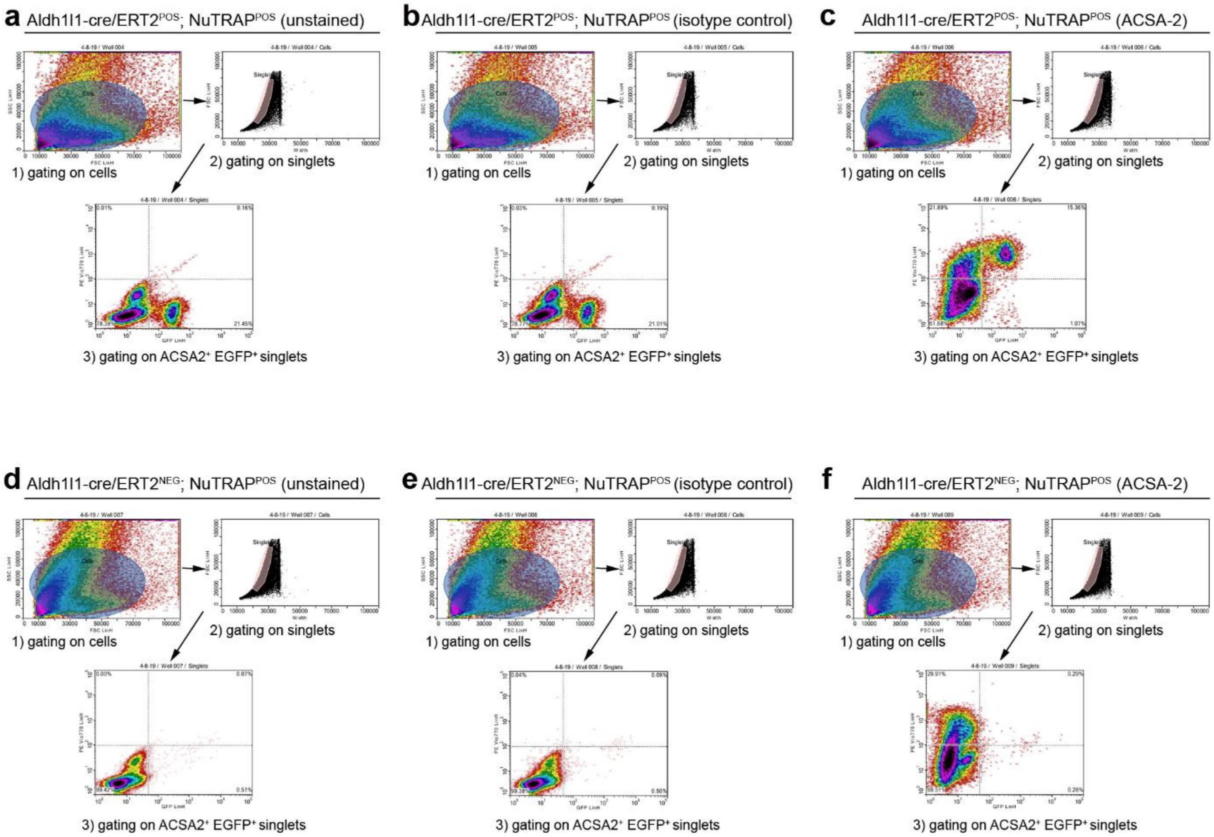

Supplementary Figure 21 Gating strategy for flow cytometry analysis of EGFP expression on ACSA-2 positive single cells in the Aldh1l1-NuTRAP model. Representative density plots from the flow cytometry results illustrate steps followed for gating EGFP$^+$ cells from ACSA-2$^+$ single cells from single-cell suspension of Aldh1l1-NuTRAP brains analyzed. Gating strategy is shown for **(a)** unstained, **(b)** isotype control-APC, and **(c)** ACSA-2-APC stained cells from the same brain cell suspension. 1) Cells are gated in the scatter range for (2) subsequent selection of single cells (singlets). 3) Using a combination of filters, singlets are further gated for ACSA-2$^+$ and EGFP$^+$ expression. Same gating strategy applied to cre negative samples (Aldh1l1cre/ERT2$^-$; NuTRAP$^+$) is represented in **(d-e-f)**.

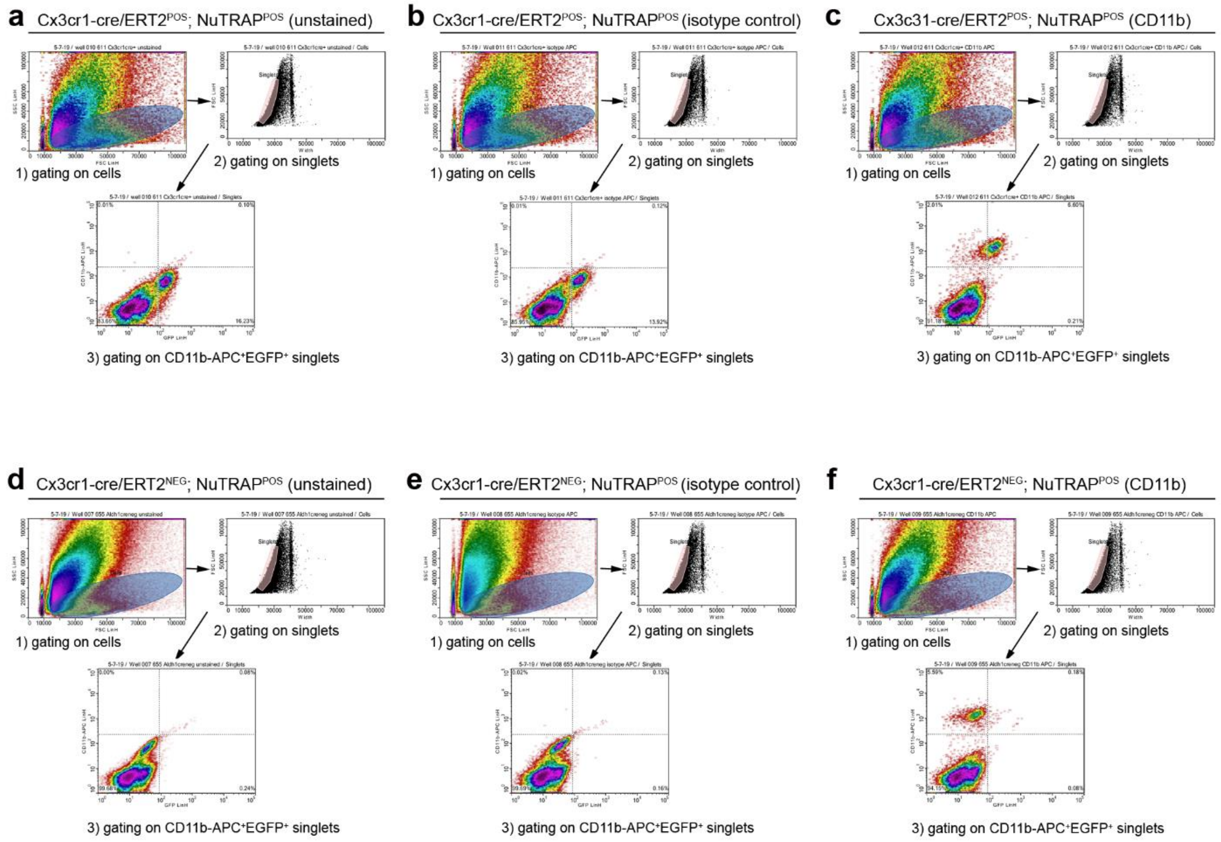

Supplementary Figure 22. Gating strategy for flow cytometry analysis of EGFP expression on CD11b positive single cells in the Cx3cr1-NuTRAP model. Representative density plots from the flow cytometry results illustrate steps followed for gating EGFP$^+$ cells from CD11b$^+$ single cells from single-cell suspension of Cx3cr1-NuTRAP brains analyzed. Gating strategy is shown for **(a)** unstained, **(b)** isotype control-APC, and **(c)** CD11b-APC stained cells from the same brain cell suspension. 1) Cells are gated in the scatter range for (2) subsequent selection of single cells (singlets). 3) Using a combination of filters, singlets are further gated for CD11b$^+$ and EGFP$^+$ expression. Same gating strategy applied to cre negative samples (Aldh1l1cre/ERT2$^-$; NuTRAP$^+$) is represented in **(d-e-f)**.

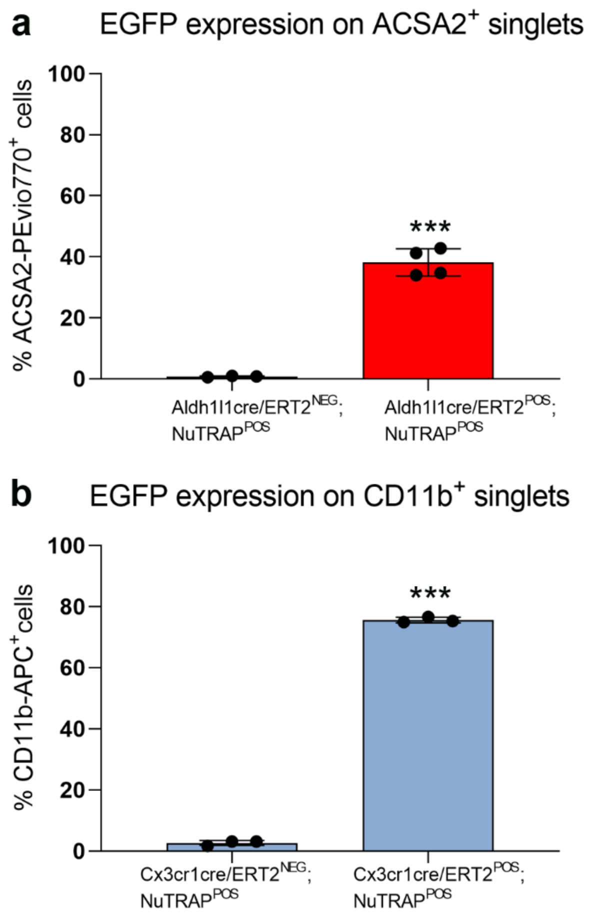

Supplementary Figure 23. a Percentage of ACSA-2 positive single cells that are EGFP+ following the gating strategy in Supplemental Figure 23. Data is expressed as mean percentage/brain sample ±SEM (n=3-4/group). **b** Percentage of CD11b positive single cells that are EGFP+ following the gating strategy in Supplementary Figure 22. Data is expressed as mean percentage/brain sample ±SEM (n=3/group). *p<0.001 by unpaired T test comparison. Note: samples are the same ones used for analysis shown in Figures 2 and 5.

gene/gene ID	Description	Taqman Gene Expression assay ID
Aldh1l1	aldehyde dehydrogenase 1 family, member L1	Mm03048957_m1
Fabp7	fatty acid binding protein 7, brain	Mm00445225_m1
Gfap	glial Fibrillary Acidic Protein	Mm01253033_m1
Elovl2	elongation of very long chain fatty acids-like 2	Mm00517086_m1
Aqp4	aquaporin 4	Mm00802131_m1
Kcnj10	potassium inwardly-rectifying channel, subfamily J, member 10	Mm00445028_m1
Cx3cr1	C-X3-C Motif Chemokine Receptor 1	Mm00438354_m1
C1qa	complement C1q A Chain	Mm00432142_m1
Gpr84	G protein-coupled receptor 84	Mm00518921_m1
Aif1	allograft Inflammatory Factor 1	Mm00479862_g1
Itgam	Integrin Subunit Alpha M	Mm00434455_m1
Eno2	enolase 2	Mm00469062_m1
Kcnb2	potassium voltage gated channel, Shab-related subfamily, member 2	Mm03057813_m1
Syt2	synaptotagmin II	Mm00436864_m1
Syt4	synaptotagmin IV	Mm01157571_m1
Npas4	Neuronal PAS Domain Protein 4	Mm01227866_g1
Mog	myelin oligodendrocyte glycoprotein	Mm01279062_m1
Neu4	sialidase 4	Mm00620597_m1
Opalin	oligodendrocytic myelin paranodal and inner loop protein	Mm00463365_m1
Il1a	interleukin 1 Alpha	Mm00439620_m1
Il1b	interleukin 1 Beta	Mm00434228_m1
Tlr2	toll- like receptor 2	Mm00442346_m1
Tlr4	toll- like receptor 4	Mm00445274_m1
Myd88	myeloid differentiation primary response gene 88	Mm01351743_g1
Tnf	tumor necrosis factor	Mm00443260_g1
Gapdh	Glyceraldehyde-3-Phosphate Dehydrogenase	4352661
Hprt	hypoxanthine guanine phosphoribosyl transferase	Mm01324427_m1

Supplementary Table 1. Taqman gene expression assays used in qPCR analyses of the study. qPCR was performed with gene-specific primer probe fluorogenic exonuclease assays (TaqMan, Life Technologies, Waltham, MA) and the QuantStudio™ 12K Flex Real-Time PCR System (Applied Biosystems). *Hprt* or *Gapdh* were used as an endogenous control.
