# Supplementary material for: Inducible cell-specific mouse models for paired epigenetic and transcriptomic studies of microglia and astroglia
Source: Commun Biol. 2020 Nov 19;3:693. doi: 10.1038/s42003-020-01418-x (PMC7678837; doi:10.1038/s42003-020-01418-x)
Supplement: Supplementary file 3 — Description of Additional Supplementary Files [file 42003_2020_1418_MOESM3_ESM.pdf]

Description of Additional Supplementary Files

Title: Supplementary Data 1.

Description: Gene lists from Figure 3. Cell type specific maker gene lists were generated from re-analysis of published data of immunopurified and high throughput single cell data from mice. Published lists were filtered first by mean enrichment score of ≥3.5 and secondly to remove any genes that appeared on lists for multiple cell types. Comparisons of astrocyte gene enrichment in this study to previously published Aldh1l1-RiboTag and Gfap-TRAP were performed by downloading raw fastq files and processing the files through StrandNGS, as described in Methods.

Title: Supplementary Data 2.

Description: Genes lists from Figure 6. Cell type specific maker gene lists were generated from re-analysis of published data of immunopurified and high throughput single cell data from mice. Published lists were filtered first by mean enrichment score of ≥3.5 and secondly to remove any genes that appeared on lists for multiple cell types. Comparisons of microglial gene enrichment in this study to previously published Cx3cr1(Jung)-RiboTag and Cx3cr1(Litt)-TRAP were performed by downloading raw fastq files and processing the files through StrandNGS, as described in Methods.

Title: Supplementary Data 3.

Description: Primers used for genotyping of Aldh1l1- NuTRAP and Cx3cr1- NuTRAP mice (information available from Jax.org website). DNA was extracted from mouse ear punch samples for genotyping using oligo sequences available from Jax laboratories website.

Title: Supplementary Data 4.

Description: Methyl primer sequences used for BSAS assays in the study. For methylation quantitation of gene promoters, primer sets were designed based on the appropriate National Center for Biotechnology Information (NCBI) reference genome using the Methyl Primer Express v1.0 software (Thermofisher Scientific) to amplify 250-350 bp regions of interest upstream or downstream the transcription start site (TSS) from bisulfite converted DNA.

Title: Supplementary Data 5.

Description: Comprehensive information on the statistical analysis used in this study. information on the statistical analyses of the data, experimental groups, sample sizes, p values for all Figures/Supplementary Figures with graphed data in this study.

Title: Supplementary Data 6.

Description: Instrument and settings (Immunohistochemistry and imaging methods). Instrument settings for capture of raw images, as well as downstream processing (Adobe Photoshop CS5.1) of each raw image used for figure assembly in this study.

Title: Supplementary Data 7.

Description: Source data used to graph data in this study. All source data underlying the graphs presented in the main/Supplementary Figures in this study.
