# Supplementary material for: Inducible cell-specific mouse models for paired epigenetic and transcriptomic studies of microglia and astroglia
Source: Commun Biol. 2020 Nov 19;3:693. doi: 10.1038/s42003-020-01418-x (PMC7678837; doi:10.1038/s42003-020-01418-x)
Supplement: Supplementary file 11 — Reporting Summary [file 42003_2020_1418_MOESM11_ESM.pdf]

nature research

Corresponding author(s): Willard M. Freeman

Last updated by author(s): 10/07/2020

Reporting Summary

Nature Research wishes to improve the reproducibility of the work that we publish. This form provides structure for consistency and transparency in reporting. For further information on Nature Research policies, see Authors & Referees and the Editorial Policy Checklist.

Statistics

For all statistical analyses, confirm that the following items are present in the figure legend, table legend, main text, or Methods section.

n/a	Confirmed	
☐	☒	The exact sample size (*n*) for each experimental group/condition, given as a discrete number and unit of measurement
☐	☒	A statement on whether measurements were taken from distinct samples or whether the same sample was measured repeatedly
☐	☒	The statistical test(s) used AND whether they are one- or two-sided<br>*Only common tests should be described solely by name; describe more complex techniques in the Methods section.*
☐	☒	A description of all covariates tested
☐	☒	A description of any assumptions or corrections, such as tests of normality and adjustment for multiple comparisons
☐	☒	A full description of the statistical parameters including central tendency (e.g. means) or other basic estimates (e.g. regression coefficient) AND variation (e.g. standard deviation) or associated estimates of uncertainty (e.g. confidence intervals)
☐	☒	For null hypothesis testing, the test statistic (e.g. *F*, *t*, *r*) with confidence intervals, effect sizes, degrees of freedom and *P* value noted<br>*Give P values as exact values whenever suitable.*
☒	☐	For Bayesian analysis, information on the choice of priors and Markov chain Monte Carlo settings
☐	☒	For hierarchical and complex designs, identification of the appropriate level for tests and full reporting of outcomes
☒	☐	Estimates of effect sizes (e.g. Cohen's *d*, Pearson's *r*), indicating how they were calculated

Our web collection on statistics for biologists contains articles on many of the points above.

Software and code

Policy information about availability of computer code

Data collection	Commercial software: FLUOVIEW FV1000 Ver. 1.2.6.0 (Olympus) for confocal microscopy; Zen Blue ver.3.1 (Carl Zeiss Microscopy, LLC) for fluorescence microscopy image tiling; CellCapTure v5.0 RC12 (Stratedigm) for flow cytometry; CellCapTure v4.1 RC10 (Stratedigm) for flow cytometry; Expression Suite v 1.0.3 (Applied Biosystems/ThermoFisher Scentific) for qPCR; Illumina NovaSeq 6000 for RNAseq; NovaSeq 6000 (Illumina) for oxBS-seq; iSeq (Illumina) for BSAS; MiSeq (Illumina) for BSAS; Agilent 7890B GC w/ 5977B MSD and Mass Hunter Acquisition, Qualitative, Quantitative Versions: Qual:B08, Quant B09 for GC/MS.
Data analysis	Commercial software: GraphPad Prism 8 .2.0 (San Diego, California USA, www.graphpad.com) for data graphing and statistical analysis; Adobe Photoshop CS5.1 for image processing and Figure assembly; Adobe Illustrator CS5.1 for illustration generation and Figure assembly; FLUOVIEW FV1000 Ver. 1.2.6.0 (Olympus) for confocal microscopy; Zen Blue ver.3.1 (Carl Zeiss Microscopy, LLC) for fluorescence microscopy image tiling; CellCapTure v5.0 RC12 (Stratedigm) for flow cytometry; CellCapTure v4.1 RC10 (Stratedigm) for flow cytometry; Expression Suite v 1.0.3 (Applied Biosystems/ThermoFisher Scentific) for qPCR; Strand Next Generation Analysis Software (NGS) v3.1 for RNA-seq; Ingenuity Pathway Analysis (IPA) v01-12 (Qiagen Bioinformatics) for RNA-seq; Genomics Workbench 11.0 (Qiagen) for BSAS; Mass Hunter Acquisition, Qualitative, Quantitative Versions: Qual:B08, Quant B09 for GC/MS.<br>Open source software: FastQC v.0.11.8 for quality assessment of fastq files in all DNA/RNA sequencing experiments; Trimmommatic v0.35 (trimming tool) for oxBS-seq; Bismark v0.16 (alignment tool) for oxBS-seq; MethylKit R package v1.10.0 for oxBS-seq (analysis and annotation from high-throughput bisulfite sequencing); CEGX QC v0.2 for oxBS-seq (output of fastqc_data.txt files containing the conversion mean for C, mC, and hmC); R package EnrichedHeatmap v1.14.0 for oxBS-seq (to intersect methylation call files with genomic coordinates of gene lists); R software v3.6.1 (to run all R packages); Morpheus (https://software.broadinstitute.org/morpheus) for heatmap representation.

For manuscripts utilizing custom algorithms or software that are central to the research but not yet described in published literature, software must be made available to editors/reviewers. We strongly encourage code deposition in a community repository (e.g. GitHub). See the Nature Research guidelines for submitting code & software for further information.

Data

Policy information about availability of data

All manuscripts must include a data availability statement. This statement should provide the following information, where applicable:

- Accession codes, unique identifiers, or web links for publicly available datasets
- A list of figures that have associated raw data
- A description of any restrictions on data availability

Sequencing data that support the findings of this study have been deposited in GEO repository with the GSE140271 (https://www.ncbi.nlm.nih.gov/geo/query/acc.cgi?acc=GSE140271) accession code for information on oxBS-seq data (used for Figure 9 and Supplemental Figures 13 and 14). The entirety of the RNA-sequencing data is available for download in FASTQ format from NCBI Sequence Read Archive (GSE140895 and GSE140974). Supplementary imaging data are available from figshare.com with DOI: 10.6084/m9.figshare.12670895 and DOI: 10.6084/m9.figshare.12669698. All source data underlying the graphs presented in the main/Supplementary Figures are available in Supplementary Data 7. Other data that support the findings of the study are available from the corresponding author (W.M.F.) upon reasonable request.

Field-specific reporting

Please select the one below that is the best fit for your research. If you are not sure, read the appropriate sections before making your selection.

☒ Life sciences ☐ Behavioural & social sciences ☐ Ecological, evolutionary & environmental sciences

For a reference copy of the document with all sections, see nature.com/documents/nr-reporting-summary-flat.pdf

Life sciences study design

All studies must disclose on these points even when the disclosure is negative.

Sample size	No sample size calculation was performed.
Data exclusions	No data exclusions were performed except for:<br>1) sequencing data from an RNA-seq library with insufficient reads (sample #271 input Cx3cr1-NuTRAP for LPS experiment used for Figure 11, and Supplemental Figure 17). Such sample was excluded for downstream analysis.<br>2) One sample for which the analysis failed to produce data (in the input group) in Supplemental Figure 18.
Replication	Measures to verify the reproducibility of the experimental findings included biological repetition through independent experiments of the following:1) INTACT protocol to assess visual fluorescence inspection/imaging/counts of putatively cell-specific nuclei for both BSAS and WGoxBS sequencing; 2) TRAP protocol to assess both qPCR validation of cell-specific transcript enrichment/depletion by qPCR and TRAP-RNA sequencing; 3) tissue processing for IHC stainings for assessment of cell-specific recombination and flow cytometry; 4) LPS experiments for reproducibility of the model, including protocols/techniques described under items 1 and 2.
Randomization	The designation of mice for each experimental purpose was randomized and solely reliant on genotyping information of eartagged mice. Males and females were not segregated and were represented in all experiments. For LPS experiments (Figure 9 and Supplemental Figure 17) mice from the same animal cage (weaned from the same litter) were randomly assigned to LPS or PBS control group so that females and males were represented under each condition.
Blinding	When possible, blinding was practiced so that samples and analyses were processed/performed at some stage by different authors, who only received a code (mouse eartag number) for sample identification. For instance: V.A.A and K.B.B were blinded to the identity of DNA/RNA samples used for BSAS (performed by S.R.O and V.A.A), cDNA synthesis and qPCR (performed partly by K.B.B.). V.A.A and K.B.B received samples isolated by A.J.C.E (INTACT-DNA) and S.R.O (TRAP-RNA). Blinding was applied at the stage of RNAseq data running and analyses. The principal component analyses (PCA) for RNA-seq were done by the software and not influenced by any type of data labeling or selection (data collected and further analyzed with Strand software by W.M.F.).

Reporting for specific materials, systems and methods

We require information from authors about some types of materials, experimental systems and methods used in many studies. Here, indicate whether each material, system or method listed is relevant to your study. If you are not sure if a list item applies to your research, read the appropriate section before selecting a response.

Materials & experimental systems

n/a	Involved in the study
☐	☒ Antibodies
☐	☐ Eukaryotic cell lines
☐	☐ Palaeontology
☐	☒ Animals and other organisms
☐	☐ Human research participants
☐	☐ Clinical data

Methods

n/a	Involved in the study
☐	☐ ChIP-seq
☐	☒ Flow cytometry
☐	☐ MRI-based neuroimaging

Antibodies

Antibodies used

Name: catalog# (Clone, Lot), Supplier; Dilution
Rabbit anti-mCherry, #ab167453 (Lot: GR3209879-3), Abcam, Cambridge, MA; 1:500
Chicken anti-mCherry, #ab205402 (Lot:GR3271744-11), Abcam; 1:500
Chicken anti-GFAP #ab4674 (Lot:GR3281175-1), Abcam; 1:1,000
Rabbit anti-NeuN: #ab177487 (Lot: GR249899-62), Abcam; 1:200
Rat anti-CD11b, #C227 (Clone: M1/70, Lot: 0412L235), Leinco Technologies, Inc. St. Louis, MO; 1:200
Alexa Fluor594-conjugate AffiniPure Donkey anti-Rabbit IgG (H+L), #711-585-152 (Lot: 136429), Jackson ImmunoResearch Laboratories, Inc., West Grove, PA; 1:200
Alexa Fluor-647-conjugated AffiniPure Donkey anti-Chicken IgY (IgG) (H+L), #703-605-155 (Lot: 134612); Jackson ImmunoResearch Laboratories, Inc.; 1:200
Alexa Fluor-594-conjugated AffiniPure Donkey anti-Chicken IgY (IgG) (H+L), #703-585-155 (Lot: 144479); Jackson ImmunoResearch Laboratories, Inc.; 1:200
AlexaFluor-647- conjugated AffiniPure Donkey anti-Rat IgG (H+L), #712-605-150 (Lot: 143801), Jackson ImmunoResearch Laboratories, Inc.; 1:200
AlexaFluor-488 donkey anti-Rabbit IgG (H+L), #A21206 (Lot:1796375), Invitrogen by Thermo Fisher Scientific, Eugene, OR; 1:500
Anti-ACSA-2-PE-Vio770, mouse: #130-116-246 (Clone REA969, Lot:5180731099), Milteny Biotec, San Diego, CA; 1:50
Anti mouse CD11b-APC :#17-0112 (Clone M1/70, Lot: 4339583), eBioscience, San Diego, CA; 1:175
REA Control-PE-Vio770:#130-113-452 (Clone REA293, Lot: 5180508106), Miltenyi Biotec; 1:50
Rat IgG2b K Iso Control-APC, #17-4031-81 (Clone: eB149/10H5, Lot: 4298461), eBioscience; 1:175
Rabbit anti GFP, #ab290 (Lot:GR306215-1), Abcam; 1:100 for IHC and 5ug/ul for TRAP

Validation

IHC antibodies:
Preliminarily, all primary antibodies were tested on brain tissue sections of WT mice in parallel to no-primary antibody negative controls to assess for background/not specific binding of secondary antibodies. Particularly for mCherry antibody, brain samples of cre negative and WT mice side by side with cre positive samples optimally controlled for the specificity of antibody binding.
Rabbit anti-mCherry, #ab167453 [1] (Lot: GR3209879-3), Abcam, Cambridge, MA
https://www.abcam.com/mcherry-antibody-ab167453.html
Chicken anti-mCherry, #ab205402 (Lot:GR3271744-11), Abcam
https://www.abcam.com/mcherry-antibody-ab205402.html
Chicken anti-GFAP #ab4674 [2] (Lot:GR3281175-1), Abcam
https://www.abcam.com/gfap-antibody-ab4674.html
Rabbit anti-NeuN: #ab177487 [3] (Lot: GR249899-62), Abcam
https://www.abcam.com/neun-antibody-epr12763-neuronal-marker-ab177487.html
Rat anti-CD11b, #C227 [4] (Clone: M1/70, Lot: 0412L235), Leinco Technologies, Inc. St. Louis, MO
https://www.leinco.com/p/anti-mouse-cd11b-purified/
Alexa Fluor594-conjugate AffiniPure Donkey anti-Rabbit IgG (H+L), #711-585-152 (Lot: 136429), Jackson ImmunoResearch Laboratories, Inc., West Grove, PA
https://www.jacksonimmuno.com/catalog/products/711-585-152
Alexa Fluor-647-conjugated AffiniPure Donkey anti-Chicken IgY (IgG) (H+L), #703-605-155 (Lot: 134612); Jackson ImmunoResearch Laboratories
https://www.jacksonimmuno.com/catalog/products/703-605-155
Alexa Fluor-594-conjugated AffiniPure Donkey anti-Chicken IgY (IgG) (H+L), #703-585-155 (Lot: 144479); Jackson ImmunoResearch Laboratories
https://www.jacksonimmuno.com/catalog/products/703-585-155
AlexaFluor-647- conjugated AffiniPure Donkey anti-Rat IgG (H+L), #712-605-150 (Lot: 143801), Jackson ImmunoResearch Laboratories, Inc.
https://www.jacksonimmuno.com/catalog/products/712-605-150
AlexaFluor-488 donkey anti-Rabbit IgG (H+L), #A21206, Invitrogen by Thermo Fisher Scientific, Eugene, OR
https://www.thermofisher.com/antibody/product/Donkey-anti-Rabbit-IgG-H-L-Highly-Cross-Adsorbed-Secondary-Antibody-Polyclonal/A-21206
Flow cytometry antibodies: unstained cells and isotype-antibody labeled cells controls were performed for proper gating and validation of antibody specificity.
Anti-ACSA-2-PE-Vio770, mouse: #130-116-246 [5] (Clone REA969, Lot:5180731099), Milteny Biotec, San Diego, CA
https://www.miltenyibiotec.com/US-en/products/macs-flow-cytometry/antibodies/primary-antibodies/anti-acsa-2-antibodies-mouse-rea969-1-50.html#pe-vio770:30-ug-in-200-ul
Anti mouse CD11b-APC :#17-0112 (Clone M1/70 [6], Lot: 4339583), eBioscience, San Diego, CA

https://www.thermofisher.com/antibody/product/CD11b-Antibody-clone-M1-70-Monoclonal/17-0112-82
REA Control-PE-Vio770: #130-113-452 (Clone REA293, Lot: 5180508106), Miltenyi Biotec
https://www.miltenyibiotec.com/US-en/products/macs-flow-cytometry/antibodies/isotype-control-antibodies/rea-control-antibodies-rea293-1-50.html#pe-vio770:
Rat IgG2b K Iso Control-APC, #17-4031-81 (Clone: eB149/10H5, Lot: 4298461), eBioscience
https://www.thermofisher.com/antibody/product/Rat-IgG2b-kappa-clone-eB149-10H5-Isotype-Control/17-4031-81
TRAP: pull-down of magnetic bead-tagged polysomes, based on GFP detection was preliminarily controlled with cre-negative samples, which in the presence of GFP antibody yielded negligible levels of TRAP-isolated RNA in the positive fraction.
Rabbit anti GFP, #ab290 [1] (Lot:GR306215-1), Abcam
https://www.abcam.com/gfp-antibody-chip-grade-ab290.html

Citations:

1. Roh, H.C., et al. Simultaneous Transcriptional and Epigenomic Profiling from Specific Cell Types within Heterogeneous Tissues In Vivo. Cell Rep 18, 1048-1061 (2017).
2. Tewari, B.P., et al. Perineuronal nets decrease membrane capacitance of peritumoral fast spiking interneurons in a model of epilepsy. Nat Commun 9, 4724 (2018).
3. Yamazaki, Y., et al. Region- and Cell Type-Specific Facilitation of Synaptic Function at Destination Synapses Induced by Oligodendrocyte Depolarization. J Neurosci 39, 4036-4050 (2019).
4. Rappert, A., et al. CXCR3-dependent microglial recruitment is essential for dendrite loss after brain lesion. J Neurosci 24, 8500-8509 (2004).
5. Kantzer, C.G., et al. Anti-ACSA-2 defines a novel monoclonal antibody for prospective isolation of living neonatal and adult astrocytes. Glia 65, 990-1004 (2017).
6. Menendez, C.M., Jinkins, J.K. & Carr, D.J. Resident T Cells Are Unable To Control Herpes Simplex Virus-1 Activity in the Brain Ependymal Region during Latency. J Immunol 197, 1262-1275 (2016).

Eukaryotic cell lines

Policy information about cell lines

Cell line source(s)	NA
Authentication	NA
Mycoplasma contamination	NA
Commonly misidentified lines (See ICLAC register)	NA

Palaeontology

Specimen provenance	NA
Specimen deposition	NA
Dating methods	NA

☐ Tick this box to confirm that the raw and calibrated dates are available in the paper or in Supplementary Information.

Animals and other organisms

Policy information about studies involving animals; ARRIVE guidelines recommended for reporting animal research

Laboratory animals	Male and female animals of the species Mus musculus were used. Mice were purchased from the Jackson Laboratory (Bar Harbor, ME), bred, and housed in the animal facility at the OUHSC, under SPF conditions in a HEPA barrier environment. In separate breeding strategies Aldh1l1-Cre/ERT2+/wt males (stock number # 29655) and Cx3cr1-Cre/ERT2+/+ males (stock # 20940) were mated with NuTRAPflox/flox females (stock # 029899) to generate the desired progeny, Aldh1l1-cre/ERT2+/wt; NuTRAPflox/wt (Aldh1l1-cre/ERT2+; NuTRAP+) and Cx3cr1-cre/ERT2+/wt; NuTRAPflox/wt (Cx3cr1-cre/ERT2+; NuTRAP+). Mice (males and females) were ~3 months old at the time of performing experiments.
Wild animals	Study did not involve wild animals.
Field-collected samples	Study did not involve field-collected samples.
Ethics oversight	All animal procedures were approved by the Institutional Animal Care and Use Committee at the University of Oklahoma Health Sciences Center (OUHSC) and the Oklahoma Medical Research Foundation (OMRF).

Note that full information on the approval of the study protocol must also be provided in the manuscript.

Human research participants

Policy information about studies involving human research participants

Population characteristics	NA
Recruitment	NA
Ethics oversight	NA

Note that full information on the approval of the study protocol must also be provided in the manuscript.

Clinical data

Policy information about clinical studies

All manuscripts should comply with the ICMJE guidelines for publication of clinical research and a completed CONSORT checklist must be included with all submissions.

Clinical trial registration	NA
Study protocol	NA
Data collection	NA
Outcomes	NA

ChIP-seq

Data deposition

☐ Confirm that both raw and final processed data have been deposited in a public database such as GEO.

☐ Confirm that you have deposited or provided access to graph files (e.g. BED files) for the called peaks.

Data access links<br>*May remain private before publication.*	NA
Files in database submission	NA
Genome browser session<br>(e.g. UCSC)	NA

Methodology

Replicates	NA
Sequencing depth	NA
Antibodies	NA
Peak calling parameters	NA
Data quality	NA
Software	NA

Flow Cytometry

Plots

Confirm that:

☒ The axis labels state the marker and fluorochrome used (e.g. CD4-FITC).

☒ The axis scales are clearly visible. Include numbers along axes only for bottom left plot of group (a 'group' is an analysis of identical markers).

☒ All plots are contour plots with outliers or pseudocolor plots.

☒ A numerical value for number of cells or percentage (with statistics) is provided.

Methodology

Sample preparation	Halves of mouse brains were rinsed in D-PBS, sliced into 8-12 sagittal sections and placed into gentleMacs C-tubes, and processed for generation of single- cell suspensions using the Adult brain dissociation kit and gentleMacsTM Octodissociator system (#130-107-677 and #130-095-937, respectively, Milteny Biotech, San Diego, CA).The resulting cell pellet was carefully resuspended in 2.0 ml 0.1% BSA buffer (in D-PBS) and filtered through a 30 um mesh. The single-cell suspensions were then immunostained for flow cytometric analysis of EGFP+ cell populations in the brain. Preparation of flow cytometry samples:<br>a) FC Block step: prepare master mix for all samples (except unstained cells) with 23 ul 0.1%BSA/PBS + 2 ul FC block (eBioscience). Gently mix cells with 25 ul of FC block mix. Incubate at 4 °C for 15 min).<br>b) Antibody staining: Dilute antibody in 0.1% BSA/PBS and apply to cells from a) so that final concentrations are 1/50 for ACSA-2 and 1/175 for CD11b immunostanings. Gently mix cells with antibody and incubate 30 min at 4 °C in the dark.<br>c) Wash cells: Add 1ml of 0.1% BSA/PBS, mix and centrifuge 300xg, 5 min, at 4 °C. Repeat for a total of 3 washes<br>d) Fix cells: After last wash, resuspend pellet in 1ml 1% PFA/PBS). Vortex right after addition to avoid cell clump formation with PFA and store overnight in the dark at 4 °C.<br>e) Wash PFA by centrifugation and resuspend in 1ml 0.1% BSA/PBS. Cells are ready to be passed to 2m eppendorf tubes and run in the flow cytometer.
Instrument	Samples were analyzed using a Stratedigm S1400Exi flow cytometer platform (Laboratory for Molecular Biology and Cytometry Research core facility at OUHSC).
Software	Data was acquired with the CellCapTure v5.0 RC12 and v4.1 RC10 software (Stratedigm) (Laboratory for Molecular Biology and Cytometry Research core facility at OUHSC).
Cell population abundance	No sorting was performed.
Gating strategy	The gating strategy selects for single cells (singlets) in normal scatter range that are EGFP+ and subsequently ACSA-2+ (astrocytes: Figure 1 and Supplemental Figure 21) or CD11b+ (microglia: Figure 4 and Supplemental Figure 22). A 488 nm (blue) laser with 530/30 and 580/30 filter combinations was used to gate on EGFP+ cells within singlets without auto-fluorescence interference. Subsequent gating based on CD11b or ACSA-2 expression was done with 640 nm laser and 676/629 filter, or with 488 nm laser and 740 LP filter combinations, respectively. The antibodies used were anti-mouse CD11b: APC (#17-0112, clone M1/70) (eBioscience, San Diego, CA), and ACSA-2: PE-Vio770 (#130-116-246, Milteny Biotec). Isotype controls for each antibody and unstained cells were used for proper color compensation/gating adjustment (Supplemental Figures 21 and 22).

☒ Tick this box to confirm that a figure exemplifying the gating strategy is provided in the Supplementary Information.

Magnetic resonance imaging

Experimental design

Design type	NA
Design specifications	NA
Behavioral performance measures	NA

Acquisition

Imaging type(s)	NA
Field strength	NA
Sequence & imaging parameters	NA
Area of acquisition	NA

Diffusion MRI ☐ Used ☐ Not used

Preprocessing

Preprocessing software	NA
Normalization	NA
Normalization template	NA
Noise and artifact removal	NA
Volume censoring	NA

Statistical modeling & inference

Model type and settings: NA

Effect(s) tested: NA

Specify type of analysis: ☐ Whole brain ☐ ROI-based ☐ Both

Statistic type for inference (See Eklund et al. 2016): *Specify voxel-wise or cluster-wise and report all relevant parameters for cluster-wise methods.*

Correction: NA

Models & analysis

n/a	Involved in the study
☒	☐ Functional and/or effective connectivity
☒	☐ Graph analysis
☒	☐ Multivariate modeling or predictive analysis
